# Precision Delivery of Multiscale Payloads to Tissue‐Specific Targets in Plants

**DOI:** 10.1002/advs.201903551

**Published:** 2020-04-22

**Authors:** Yunteng Cao, Eugene Lim, Menglong Xu, Jing‐Ke Weng, Benedetto Marelli

**Affiliations:** ^1^ Department of Civil and Environmental Engineering Massachusetts Institute of Technology Cambridge MA 02139 USA; ^2^ Whitehead Institute for Biomedical Research Cambridge MA 02142 USA; ^3^ Department of Biology Massachusetts Institute of Technology Cambridge MA 02139 USA

**Keywords:** multiscale payload, plant engineering, precise delivery, silk fibroin, sustainable agriculture

## Abstract

The precise deployment of functional payloads to plant tissues is a new approach to help advance the fundamental understanding of plant biology and accelerate plant engineering. Here, the design of a silk‐based biomaterial is reported to fabricate a microneedle‐like device, dubbed “phytoinjector,” capable of delivering a variety of payloads ranging from small molecules to large proteins into specific loci of various plant tissues. It is shown that phytoinjector can be used to deliver payloads into plant vasculature to study material transport in xylem and phloem and to perform complex biochemical reactions in situ. In another application, it is demonstrated *Agrobacterium*‐mediated gene transfer to shoot apical meristem (SAM) and leaves at various stages of growth. Tuning of the material composition enables the fabrication of another device, dubbed “phytosampler,” which is used to precisely sample plant sap. The design of plant‐specific biomaterials to fabricate devices for drug delivery *in planta* opens new avenues to enhance plant resistance to biotic and abiotic stresses, provides new tools for diagnostics, and enables new opportunities in plant engineering.

## Graphical abstract

A silk‐based biomaterial is designed to fabricate a microneedle‐like device with controlled degradation rate in planta via tuning material composition. The device enables precise access to xylem, phloem, and other plant tissues, thus being capable of serving as a delivery tool to release cargo molecules and as a sampling tool for analysis of plants fluid.

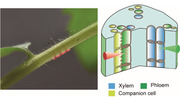

A projected world population of 9.7 billion people in 2050 may result in a 70% increase of food demand and pose a severe strain to global food security.^[^ ^1^ ^]^ To address these challenges, innovations in plant genetic engineering and precision agriculture are highly sought to enhance crop productivity, impart and/or enhance plants resistances to diseases and stresses and increase the sustainability of crop production.^[^ ^2^ ^]^ In this scenario, there is an increasing interest in the use of biomaterials and nanotechnology to plant science and crop production, provided the tremendous effects of these technologies in biomedicine (e.g., drug delivery) and microbiology. For example, nanomaterials have been used in plants as bactericides and fertilizers,^[^ ^3^, ^4^, ^5^ ^]^ microneedles have been applied on leaves to sample pathogenic bacteria^[^ ^6^ ^]^ and nanobionics has been developed to impart new function to plants’ organelles.^[^ ^7^, ^8^ ^]^ Nonetheless, the use of biomaterials and drug delivery principles to engineer the precise deployment of payloads in plants has been largely overseen. This has also resulted in limited technical capability in dealing with diseases that target plant vasculature (e.g., phloem‐ or xylem‐restricted bacteria^[^ ^9^ ^]^) and is a limiting factor in plant engineering, where nanoparticles are delivered to plant tissues by complex and inefficient methods. The most commonly used delivery methods for plants are foliar spray, root application, and trunk injection/petiole feeding.^[^ ^5^ ^]^ Although foliar spray and root application are easy to implement, they suffer from significant material loss and low efficiency due to plant's barrier tissues such as cuticle and epidermis. Trunk injection and petiole feeding overcome the challenges caused by plant barrier tissues by damaging these barriers mechanically and accessing vasculature directly. They have a higher delivery efficiency and can be used to deliver large amount of payloads. However, they are suitable for large, woody plants due to their invasive application process. Valuable and labile payloads are also not suitable to be delivered by these methods. Foliar infiltration and pressurized bath infusion^[^ ^8^ ^]^ widely used in lab also have a low delivery efficiency since most materials are left in leaves' intercellular space.

Silk fibroin (derived from *Bombyx mori*) has been extensively studied as a technical material in a wide range of fields including drug delivery and regenerative medicine,^[^ ^10^ ^]^ optoelectronics,^[^ ^11^ ^]^ and food coatings^[^ ^12^ ^]^ due to its unique properties that include nontoxicity (degradation into amino acids), mechanical robustness, tunable degradation via hydrolysis, preservation of heat‐labile payloads, and ease of fabrication. In biological sciences, the structural protein has been investigated for drug delivery as it can be fabricated into implantable devices that preserve and release payloads in vivo while not providing an adverse reaction upon implantation.^[^ ^13^ ^]^ Silk fibroin degradation in host human tissues can be modulated by controlling the protein polymorphism, i.e., the amount of β‐sheets present in the end‐material, as more ordered molecular structures are more resistant to proteolytic degradation.^[^ ^14^ ^]^ These features are attractive also for the design of a plant‐specific biomaterial for drug delivery. However, limited free water and low concentration of proteases in plant sap fluid result in prolonged silk fibroin stability and limited release of cargo molecules.^[^ ^15^ ^]^ To overcome these challenges, we engineered a new biomaterial based on silk fibroin that was formatted in a device capable of delivering a variety of payloads ranging from small molecules to large proteins into specific loci of various plant tissues.

Optimization of material's mechanical robustness and solubility was controlled by tuning the relative amount of hydrophobic/hydrophilic domains and enabled the design and fabrication of an array of injectors (namely phytoinjector) capable of targeting plant vasculature by penetrating plant dermal and ground tissues. The dimensions of tissue‐specific phytoinjectors were determined by histological analysis of the target tissue. Using specific phytoinjectors, payloads (ranging in size from small molecules to large proteins) were deployed in tomato plant xylem and phloem and their transport from source to sink was observed and modeled. *Agrobacterium*‐loaded phytoinjectors also showed gene transfer to and expression in tobacco shoot apical meristem (SAM) and in leaves at various stages of growth. Tuning of material composition also enabled the fabrication of a device to sample xylem sap.

Silk fibroin heavy chain (≈390 kDa) is composed of 12 large, hydrophobic amino acid domains that amount for more than 75% of the protein and that are linked by 11 short, hydrophilic spacers (**Figure** **1**a). Preliminary investigations using silk fibroin showed limited payload release in xylem and phloem saps. Partial insolubility in plant sap may negatively affect sap flow in xylem and phloem by vascular blockage. To overcome these challenges, we used a top‐down synthetic approach to increase the hydrophilic content of the silk end‐material and decrease the size of the protein biodegradation byproducts by extracting hydrophilic silk fibroin‐derived polypeptides (Cs) (Figure 1a; Figure S1, Supporting Information).^[^ ^16^ ^]^ *α*‐chymotrypsin allows to extract silk fibroin‐derived soluble peptides (Cs)^[^ ^16^ ^]^ that can be mixed with silk fibroin water suspensions, yielding a more hydrophilic silk material that also disrupts the hydrophobic effect‐derived aggregation of silk molecules in nanomicelles. In aqueous suspension, Cs does not show noticeable influence on silk nanomicelle size (Figure S1, Supporting Information). In terms of composition, Cs is composed of negatively charged peptides with a molecular weight of 2–10 kDa (Figure S1, Supporting Information) and a primary structure that accounts for only 10–15% of hydrophobic amino acids. As a result, Cs is highly soluble but also yields very brittle materials, which makes it unsuitable (as a stand‐alone entity) for the fabrication of payload delivery devices. However, Cs can be blended with silk fibroin with the weight ratio of the two biopolymers being tuned to modulate fundamental biomaterial end‐properties such as solubility, degradation, mechanical strength, nanomicelle size, and preservation of payloads. Cs is incorporated in silk materials during the assembly process, when hydrogen bonds between silk nanomicelles and water are replaced with intermolecular hydrogen bonds. During this step, nanomicelles coalesce and form a monolithic material. Cs would then participate in this assembly process as it is made by a portion of the silk fibroin primary structure. However, being of smaller molecular weight, the incorporation of Cs results in the weakening of the interactions/entanglement between large silk fibroin molecules, ultimately enhancing material disassembly upon exposure to water. The intermolecular and intramolecular interaction of hydrophobic amino acid domains may also be weakened. To further explore this mechanism, we have conducted several investigations of silk fibroin–Cs interactions both in water suspension and in solid, monolithic materials (i.e., film format), which has been reported in Supporting Information. Materials characterization was also accomplished to identify the optimal composition for payload delivery into plants. In the manuscript, we denote a Cs 20%–silk fibroin 80% dry weight mixture as Cs_20_SF_80_. SF refers to pure silk fibroin.

**Figure 1:**
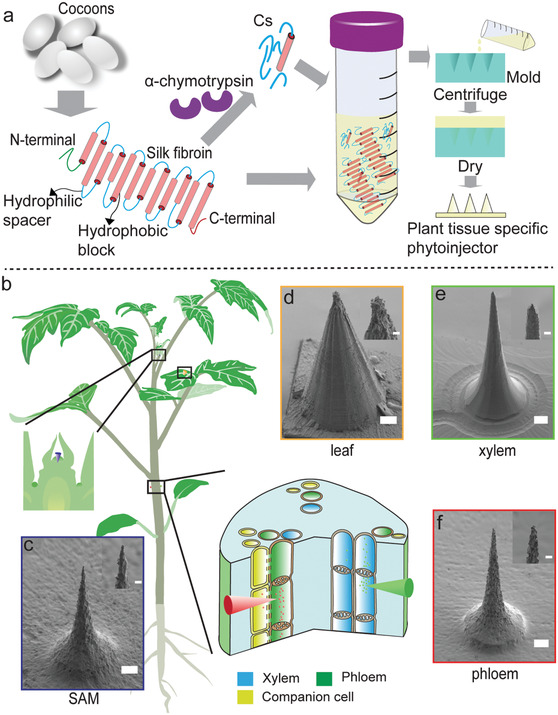
Material and device design for multiscale, multitissue precise delivery of payloads in plants. a) Material design. Silk materials were engineered to perform in plants. Silk fibroin is first extracted from *Bombyx mori* cocoons; the 390 kDa heavy chain is composed of 12 hydrophobic blocks (red cylinders) staggered by 11 hydrophilic spacers (blue lines). By using α‐chymotrypsin, the hydrophilic spacers (Cs) can be extracted. The final material is a blend of Cs and silk fibroin, which is fabricated intoplant tissue specific phytoinjectors via PDMS molds. b) Silk fibroin materials can be fabricated in phytoinjectors of desired size and shape for precise payloads delivery in different plant tissues. In the schematic, injection in foliar tissue, shoot apical meristem, and plant vasculature are represented. In particular, the green and red injectors indicate delivery to xylem and phloem, respectively. The left inset indicates delivery to shoot apical meristem. c–f) Scanning electron images of phytoinjectors designed for delivery to SAM, leaf, xylem, and phloem, respectively. The inlets show the injectors tips. Scale bar: 100 µm, scale bar of inlet: 20 µm.

Cs–silk fibroin biomaterials were characterized according to the following properties: solubility, nanomicelle size when resolubilized, conformation, viability of preserved labile payloads, and mechanical robustness. Solubility in simulated sap increases dramatically with increased Cs content (**Figure** **2**a). Compared to silk fibroin (89.8 mg mL^−1^), the maximum concentration of Cs_20_SF_80_ in suspension is two times higher (184.1 mg mL^−1^), while the concentration of pure Cs at saturation is five times higher (441.3 mg mL^−1^). Nanomicelle size of resuspended Cs_20_SF_80_ has no significant difference from that of resuspended silk fibroin (Figure S1, Supporting Information). The protein structure was investigated both in suspension by circular dichroism (CD) and in solid state (film form) by attenuated total reflectance Fourier transform infrared spectroscopy (ATR‐FTIR), Raman spectroscopy, thermogravimetric analysis, and differential scanning calorimetry (DSC). CD spectra of silk fibroin show a strong negative peak at 196 nm, indicating large amounts of random coils and a weak negative peak at 216 nm, distinctive of limited amounts of *β*‐sheets.^[^ ^17^ ^]^ Pure Cs shows a strong negative peak at 190 nm and a weak negative peak at 216 nm, indicating the presence of *β*‐turns and *β*‐sheets, respectively (Figure 2b). No noticeable conformation changes occur due to the blending of Cs and silk fibroin. FTIR spectra also show little difference and Amide I absorbance is dominated by a resonance centered at 1645 cm^−1^ (Figure S2, Supporting Information) that is characteristic of random coils.^[^ ^18^ ^]^ Incorporation of increasing concentrations of Cs in the blends did not result in a change of β‐sheet content (Figure S2, Supporting Information), suggesting that Cs did not drive a random coil to β‐sheet transition during silk fibroin assembly. The slight increase of turns with Cs content increase may attribute to the intrinsic molecule properties of Cs. Analysis of the Amide bands in Raman Spectra (Figure S3, Supporting Information) indicates that Cs does not hinder polymorphic changes of the structural protein.^[^ ^19^ ^]^ The difference of decomposition temperature of Cs (180 °C), SF (225 °C), and Cs_20_SF_80_ (205 °C) (Figure S4, Supporting Information) indicates weakened molecular interaction between silk fibroin by Cs, which agrees with DSC results (Figure S4, Supporting Information).^[^ ^20^ ^]^

**Figure 2:**
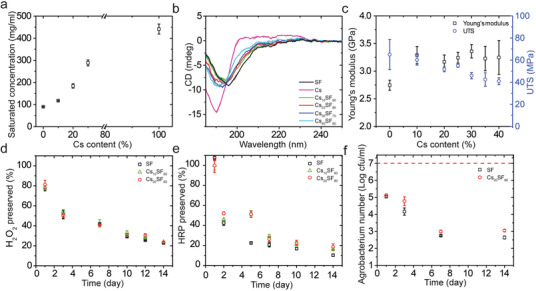
Material characterization of engineered silk material for *in planta* applications. a) Solubility of Cs–silk fibroin blends (Cs*_xx_*SF*_yy_*) in simulated sap fluid. Cs dramatically increases the solubility of CsSF blends, resulting in materials that can easily biodegrade in a sap‐like environment. b) CD spectra of CsSF blends with various Cs content. c) Mechanical properties of CsSF films with various Cs content under tension. d) Hydrogen peroxide preservation in SF, Cs_10_SF_90_, and Cs_20_SF_80_. e) HRP preservation in SF, Cs_10_SF_90_, and Cs_20_SF_80._ f) *Agrobacterium* preservation in SF and Cs_20_SF_80_. Data are mean ± s.d (*n* is at least 3).

Hydrogen peroxide was selected as a small molecule used for labile payload preservation due to its significant metabolic functions, which include lignification, ABA signaling in guard cells, programmed cell death, and pathogen response.^[^ ^21^ ^]^ Based on the mechanical properties of the CsSF blends (Figure 2c, discussed later), Cs content was limited to 20% or less. Hydrogen peroxide was well preserved in SF, Cs_10_SF_90_, and Cs_20_SF_80_, showing no significant differences among the three materials (Figure 2d). In Cs_20_SF_80_ films, 81% and 50% of entrapped hydrogen peroxide was preserved at day 1 and 3 postdrying, respectively. At day 14, 24% of hydrogen peroxide was preserved, and this preservation window can be extended beyond three weeks. This strong oxidant is trapped in the Cs–silk fibroin matrix without chemical reactions, similarly to the presence of free water in the material (Figure S5, Supporting Information).^[^ ^22^ ^]^ Horseradish peroxidase (HRP) was used as a model to test preservation of enzymes and proteins. In this case, Cs_20_SF_80_ blends enhanced the preservation of the enzyme, which had 51% and 19% residual catalytic activity at days 5 and 14, respectively. To assess preservation of bacteria in Cs–silk fibroin blends, *Agrobacterium tumefaciens* was added to SF and Cs_20_SF_80_. The number of live bacteria preserved in dried SF and Cs_20_SF_80_ showed a 2‐log reduction after 24 h, due to the drying process. At day 7, a further 2‐log decrease in bacteria viability was measured. Cs_20_SF_80_ shows a slightly improved performance in preserving *Agrobacterium* than SF (Figure 2f). The feasibility of injecting CsSF mixtures in plants was first explored by investigating their mechanical properties via uniaxial tensile strength and nanoindentation measurements (Figure S6, Supporting Information). SF Young's modulus was 2.75 ± 0.09 GPa (Figure 2c), which is in the range of previously reported measurements.^[^ ^23^ ^]^ The addition of Cs into silk fibroin materials enhances the Young's modulus by more than 15% but at the cost of ductility, which further confirms our proposed mechanism of interaction between Cs and silk fibroin. Nanoindentation results also indicate that reduced modulus increases with increasing Cs content from 0% up to 40%.

Payload release profiles of silk fibroin constructs in sap fluid follows a *Super Case II* mechanism (see Figure S7 and Table S2, Supporting Information). To demonstrate targeted payload delivery to xylem and phloem, we combined Cs_20_SF_80_ with replica‐molding to fabricate phytoinjectors of different sizes. To identify potential modes of entry to plant vasculature, we prepared and analyzed histological samples of tomato (*Solanum lycopersicum L*.) stem and petiole. We used tomato as the working model because of the well‐defined structure of the vasculature, presence of compound leaves with long petiole (Figure S8, Supporting Information), and importance as crop. The penetration depth, defined as the segment between the vasculature and the epidermis, is in the range of 840–1040 µm and 707–925 µm for xylem and phloem, respectively, and depends on the diameter of petiole (Figure S8, Supporting Information). The reported diameters of xylem and phloem are of the order of tens and hundreds of µm, respectively.^[^ ^24^ ^]^ Based on these parameters, phytoinjectors were designed with a tip diameter smaller than 35 and 10 µm for xylem and phloem, respectively (Figure 1b; Figure S8, Supporting Information). Resuspended Cs_20_SF_80_ has a particle size of 3–7 nm (Figure S1, Supporting Information), which suggests that it can be transported in xylem through the pit membrane (pore size 5–420 nm^[^ ^25^ ^]^) and in phloem through the sieve plate (pore size 610 ± 150 nm in *S. lycopersicum* ^[^ ^26^ ^]^). Phytoinjectors exhibit appropriate mechanical robustness for injection to various tissues of tomato plant, tobacco plant and citrus tree (**Tables** **1** and **2**; Figure S9, Supporting Information). To investigate payload delivery *in planta*, each payload was loaded to phytoinjectors at the point of material assembly before drying. Rhodamine 6G and 5(6)‐carboxyfluorescein diacetate were incorporated into phytoinjectors to target phloem and xylem, respectively, and injected in tomatoes’ petioles (**Figure** **3**a). Petiole cross‐section showed that the phytoinjectors reached the vasculature (Figure 3b). Histological analysis also corroborated these findings (Figure 3c). The injected petioles were sliced along the transverse section downstream and upstream at various distances from the injection site to investigate the presence of the delivered dyes. For phloem injections, rhodamine 6G was transported further downstream (i.e., from leaf to root for a mature leaf, >3.3 cm) than upstream (≈0.3 cm) from the injection site. This result is in accordance with reported translocation in phloem for mature leaves^[^ ^24^, ^27^ ^]^ (Figure 3d) and indicates that phytoinjectors successfully deployed payloads in the phloem that were translocated along the vascular tissue. In xylem, transport analysis was conducted by tangential sectioning of the stem (Figure 3e). Analysis of 5(6)‐carboxyfluorescein diacetate indicated that the molecule was transported more than 7 cm downstream (i.e., from root to leaves), and 1 cm upstream from the injection site. Upstream transport was likely the result of pure diffusive phenomena. The longer transport detected in the xylem when compared to phloem may be attributed to a more efficient deployment in its conduits, which also facilitated analysis conditions due to their larger diameter and smaller background noise of green fluorescence. To quantify payloads transport, we integrated the fluorescence intensity in Figure 3e. The normalized intensity distribution evolves spatially and temporally (Figure 3f). Notably, the dye was also transported along the radial system of the vasculature. However, in this study we focus on longitudinal material transport only, thus ignoring the radial phenomena by integration, which results in a simplified 1D problem (see Supporting Information).

**Table 1:** Tip breaking force of phytoinjectors

	Cs_20_SF_80_ phloem phytoinjector	SF phloem phytoinjector	Cs_20_SF_80_ xylem phytoinjector	SF xylem phytoinjector
Tip breaking force [N]	0.142 ± 0.022	0.151 ± 0.015	0.392 ± 0.043	0.400 ± 0.080

**Table 2:** Plant tissue penetration force by a xylem phytoinjector

Plant	Tomato			Tobacco		Citrus	
Tissue	Stem	Petiole	Leaflet	Petiole	Leaf	Branch	Leaf
Penetration force [mN]	30.4 ± 10.1	24.0 ± 8.4	5.2 ± 0.5	23.3 ± 1.0	9.1 ± 2.7	32.2 ± 4.6	22.8 ± 1.0

**Figure 3:**
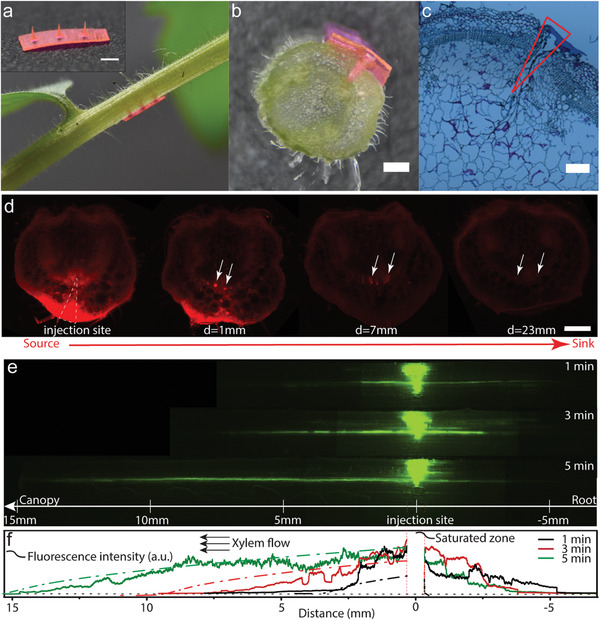
Payload delivery to tomato plant vasculature system. a) A tomato plant injected in the petiole by an array of phytoinjectors loaded with rhodamine 6G. The phytoinjector array is showed on top left. Scale bar: 1 mm. b) Cross‐section of the injection site, depicting a phytoinjector that reaches tomato petiole vasculature system. Scale bar: 500 µm. c) Bright field image of a histological section of stem's cross‐section at injection site. Scale bar: 200 µm. d) Fluorescent microscope images showing rhodamine 6G delivered to and transported in phloem, from source to sink. The red spots highlighted by white arrows point to rhodamine 6G in phloem. Scale bar: 500 µm. e) Image assembly of fluorescent images showing 5(6)‐carboxyfluorescein diacetate delivered to and transported in xylem, from roots to canopy, 1, 3, and 5 min postinjection. f) Corresponding fluorescent intensity depicting 5(6)‐carboxyfluorescein diacetate distribution along xylem (1, 3, and 5 min postinjection, respectively). Red dashed line highlights the saturated zone due to residue of the phytoinjetor, which is removed from experimental data. Black dot line is the background. Solid curves are experimental data while dash dot lines with the same color are corresponding model simulation.

There are numerous examples of small molecules, macromolecules, and bacteria that have been delivered in leaf tissue and roots to modify plants’ genome, boost photosynthesis, and act as pesticide or fertilizer.^[^ ^4^ ^]^ Injection in the stem (or trunk) has also been performed to deliver antibiotics, pesticides, and nutrients.^[^ ^28^ ^]^ Here, to provide a proof of concept that silk‐based phytoinjectors can precisely orchestrate the deployment of different payloads in plant vasculature, we have designed a multireagents delivery system that enables the well‐known luciferin–luciferase bioluminescent reaction^[^ ^8^, ^29^ ^]^ in plant vasculature

(1) $$\begin{array}{ccc} & & \mathrm{Luciferin}+\mathrm{ATP}+O_{2}\;\overset{Mg^{2+}}{\underset{\mathrm{Luciferase}}{\to }}\mathrm{Oxyluciferin}+\mathrm{AMP}+\mathrm{PPi}\\ & & \;+\;CO_{2}+hv \end{array}$$

where AMP is adenosine monophosphate, ATP is adenosine triphosphate, PPi is inorganic pyrophosphate, and *hv* is light. We deployed a bioluminescent system in plant vasculature as a model for the complex biochemical interactions occurring during transport of hormones, signaling molecules, and peptides. We chose to apply the phytoinjectors in petiole vasculature near the terminal leaflet to facilitate observation and imaging due to the limited amount of payloads delivered. At first, we deployed luciferin in the petiole's xylem while the other reagents were delivered by foliar infiltration to the leaf. The leaf tissues downstream the injection site showed luminescence (**Figure** **4**a), indicating the occurrence of the reaction, thus the delivery of luciferin and mobility of small molecules through the vasculature into ground tissue. Interestingly, no noticeable luminescence was observed from main veins, suggesting impermeability of vein structure to some reagents, likely luciferase due to its size. Luciferin and luciferase were then loaded to different phytoinjectors and injected to the same petiole (Figure 4b), while the other reagents were infiltrated in the leaf ground tissue. Though faint, luminescence was detected in the vein of the leaf (Figure 4b), indicating the delivery of multi reagents as well as a large protein via phytoinjectors.

**Figure 4:**
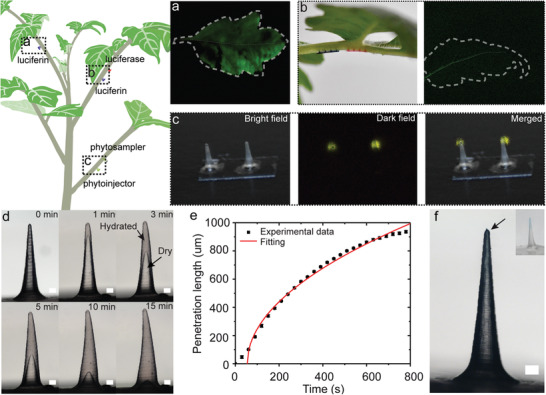
Delivery and sampling of biomolecules in xylem. a) Delivery of luciferin into the petiole xylem; by providing external luciferase, ATP, and Mg^2+^, the whole leaf emits light (exposure time 30 s, image adjusted for display purpose). b) Two arrays of phytoinjectors loaded with different payloads (luciferin for blue injectors and luciferase for red ones, blue and red here are only for display purpose) targeting petiole's xylem concurrently. By providing external ATP and Mg^2+^, the leaf vein emits light (exposure time 120 s, image adjusted for display purpose). c) Sampling of luciferin and Mg^2+^ delivered to petiole xylem by Cs_20_SF_80_ phytoinjectors using an SF phytosampler (exposure time 30 s for dark field). d) Swelling of and water movement in a phytosampler injected into agar gel indicating the possible use to sample plant fluids. e) Corresponding water penetration length with time. f) A phytosampler injected into toluidine blue agar gel becomes blue in 1 min. Data are mean ± s.d (*n* = 3).

Leveraging the polymorphic nature of silk materials, it was also possible to design water insoluble devices that reswell when exposed to sap fluid and can be removed post‐injection. Such devices are here named phytosampler as they can be used to sample sap fluids. Since partial dissolution of the phytosampler is undesired, we used pure silk fibroin as fabrication material. The efficacy of the phytosampler was assessed by deploying it in the xylem downstream to a phytoinjector loaded with luciferin and Mg^2+^. Upon sampling, the phytosampler was exposed to the reaming reagent necessary for the bioluminescent reaction to occur. Generation of light indicated the successful sampling of luciferin and Mg^2+^ from the xylem (Figure 4c). The dislocation of phytoinjecor tip and luminescence spot in merged image is likely due to diffusion of luciferin into the solution drop of reagents and deformation of silk fibroin substrate when exposed to the reagents. Reswelling of the phytoinjectors and diffusion of metabolite and catabolite in silk phytosampler was modeled with a Lucas–Washburn equation^[^ ^30^ ^]^ (Figure 4e) by investigating the diffusion of water and dyes like toluidine blue in the device (Figure 4d,f), although poroelastic models^[^ ^31^ ^]^ could also be applied to take into account for the relaxation of the transient response of silk materials during reswelling.

To assess targeted delivery of live microorganisms into plant tissues, we loaded *Agrobacterium tumefaciens* with a pEAQ‐HT vector containing *gfp* gene into Cs_20_SF_80_ phytoinjectors, using tobacco (*Nicotiana benthamiana*) as a model plant. *A. tumefaciens* has been widely used as a powerful gene transformation vehicle in plant genetic engineering to optimize the crop production of the desired products, such as drugs or proteins.^[^ ^32^ ^]^ *A. tumefaciens*‐mediated genetic transformation can target: 1) developing tissues^[^ ^33^ ^]^, 2) inflorescences via floral dipping, or 3) leaves via foliar infiltration. We targeted SAMs, young growing leaves, and mature leaves. The phytoinjector dimensions were modified to optimize payload delivery via SAM injection and leaf injection (Figure 1f). At two weeks post‐injection (when the SAM became a leaf), the leaves were harvested. Although all leaves exhibited GFP‐induced fluorescence, the spatial distribution of GFP synthesis differed. Leaves derived from treated SAMs exhibited scattered GFP fluorescence across the leaf when excited with blue light (**Figure** **5**a,ii). Using fluorescence microscopy, GFP expression was detected in multiple spots situated across the entire leaf (Figure 5a,iii), indicating successful gene transfer in mesophyll cells. The scattered distribution of these cells may result from cell divisions and subsequent growth of SAM cells. Since some (but not all) of the SAM cells that were directly in contact with *A. tumefaciens* (released from the Cs_20_SF_80_ phytoinjector) demonstrated gene transfer, we hypothesize that GFP‐expressing cells were isolated by non‐GFP‐expressing cells during leaf growth. The young leaves grew in the two weeks postinjection, and GFP fluorescence in the form of lines or scattered spots situated was observed away from the injection site (Figure 5b,iii). This differs from what was observed in mature leaves, where GFP expression was limited to cells that are close to the injection site (Figure 5c,iii). The limited degree of gene transfer in mature leaves suggests that *A. tumefaciens* has little to no mobility upon release in the leaf. This is validated by foliar infiltration, where GFP expression in cells is generally limited to the area directly accessible to *A. tumefaciens*. In growing young leaves, cells can divide and grow, so GFP‐expressing cells form lines and scattered spots, depending on the geometrical growth of the leaf. Altogether, these results demonstrate that *A. tumefaciens*‐mediated gene transfer to plant tissues can be achieved using Cs_20_SF_80_ phytoinjectors.

**Figure 5:**
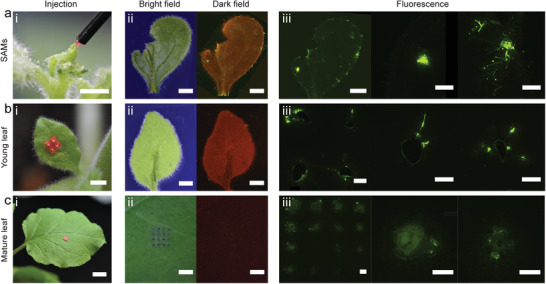
*Agrobacterium*‐mediated gene transfer to shoot apical meristem and leaves. a) *Agrobacterium* delivered to the shoot apical meristem. (i) Shoot apical meristem injected by a phytoinjector loaded with agrobacteria (rhodamine 6G was also loaded for display purpose). (ii) Bright and dark field images of the leaf from the shoot 2 weeks after the injection. Bright green spots in dark field indicating GFP expressed in leaf cells are distributed across the whole leaf. (iii) Fluorescent microscope images of the leaf in (ii). *Agrobacterium* delivered to b) a young leaf and c) a mature leaf. (i–iii) Images when injected, bright and dark field images 2 weeks after injection, and fluorescent microscope images of the injected area on leaves. GFP is observed away from the injection site in a young leaf due to tissue growth, while it expressed only at the injection site in a mature leaf. Scale bar 2 mm for (i) and (ii). 500 µm for (iii). Exposure time, bright field 20 ms, dark field 5 s.

Microneedles have been previously reported for pain‐free transdermal drug delivery and vaccination.^[^ ^34^ ^]^ In this study, we used principles of biomaterial design to fabricate phytoinjector and phytosampler devices to deliver cargo molecules to plants and to investigate material transport phenomena in plant vasculature. Our current design enables the delivery of tens of ng of cargo molecules per injector and thus cannot be used to deliver sufficient amounts of macronutrients for plants (Table S3, Supporting Information). However, there is a large variety of payloads that function in plants at quantities that can be delivered with the current phytoinjector setup (Tables S3 and S4, Supporting Information). Examples are: plant hormones, micronutrients, small interfering RNA (siRNA), and self‐replicating microorganisms. Injection and silk degradation appeared to not compromise the functionality of both xylem and phloem and did not noticeably affect plant health, despite the formation of scar tissue around the injection site at day 14 postinjection (Figure S10, Supporting Information). Immediate material degradation to nanometer‐scale particles and the general bioinert nature of silk fibroin may, in fact, have resulted in a rapid recovery to physiological function upon flow disruption, with no evident adverse reaction to plant health at day 7 postinjection (Figure S10, Supporting Information) and on sap flow (Figure 3). Future studies are however necessary to investigate plant responses to the injection, e.g., through studying Ca^2+[^ ^35^ ^]^ and jasmonic acid signaling.^[^ ^36^ ^]^ The precise targeting of phloem here described may also open the door to future applications in systemic signaling molecules release *in planta*,^[^ ^37^ ^]^ which is currently not possible. Accessing the phloem has in fact always been a technological challenge that is currently addressed using Pico gauge^[^ ^38^ ^]^ or by severing an aphid stylet during feeding.^[^ ^39^ ^]^ Precise injection in SAM also enabled the modification of plant genotype to induce expression in the current generation. We have also expanded the function of silk‐based phytoinjectors to achieve analyte sampling from plant vasculature. Potential sampling applications of insoluble phytoinjectors include detection of early‐stage phloem‐ and xylem‐limited pathogens, natural plant response to environmental cues, and engineered plant response to user‐defined cues. In conclusion, the design of plant‐specific biomaterials to fabricate devices for drug delivery *in planta* opens new avenues to enhance plant resistance to biotic and abiotic stresses, provides new tools for diagnostics, and enables new opportunities in plant engineering.

## Experimental Section

Experimental details are available in the Supporting Information.

## Conflict of Interest

The authors declare no conflict of interest.

## Supporting information

Supporting Information
