# Supplementary material for: Precision Delivery of Multiscale Payloads to Tissue‐Specific Targets in Plants
Source: Adv Sci (Weinh). 2020 Apr 22;7(13):1903551. doi: 10.1002/advs.201903551 (PMC7341084; doi:10.1002/advs.201903551)
Supplement: Supplementary file 1 — Supporting Information [file ADVS-7-1903551-s001.pdf]

Copyright WILEY-VCH Verlag GmbH & Co. KGaA, 69469 Weinheim, Germany, 2019.

Supporting Information

Precision Delivery of Multi-Scale Payloads to Tissue-Specific Targets in Plants

*Yunteng Cao, Eugene Lim, Menglong Xu, Jing-Ke Weng, Benedetto Marelli**

Y. Cao, Dr. E. Lim, Prof. B. Marelli
Department of Civil and Environmental Engineering, Massachusetts Institute of Technology, Cambridge, MA, 02139, USA.
E-mail: bmarelli@mit.edu
Dr. M. Xu, Prof. J.K. Weng
Whitehead Institute for Biomedical Research, Cambridge, MA, 02142, USA.
Prof. J.K. Weng
Department of Biology, Massachusetts Institute of Technology, Cambridge, MA, 02139, USA.

Analysis of interaction between Cs and silk fibroin

Cs is family of highly water soluble, negatively charged peptides extracted from silk fibroin heavy chain with a MW between 2-10kDa (Figure S1) and a primary structure that accounts for only 10-15% of hydrophobic amino acids. We used Cs to enhance silk fibroin solubility for in planta application to build on the biodegradability and non-toxic nature of silk-based materials. Silk fibroin used in this study has an average MW of 100-150 kDa (Figure S1) and we fabricated blends with a weight ratio between 0 to 40% Cs. By molarity, this means that in the blends, the number of Cs molecules is larger than the amount of silk fibroin. For example, for $Cs_{20}SF_{80}$ blends, we have roughly five times more Cs molecules than silk fibroin ones in the final material. Cs is incorporated in silk materials during the assembly process, when hydrogen bonds between silk nanomicelles and water are replaced with intermolecular hydrogen bonds. During this step, nanomicelles coalesce and form a monolithic material. Cs would then participate in this assembly process as it is made by a portion of the silk fibroin primary structure. However, being of smaller MW, the incorporation of Cs results in the weakening of the interactions/entanglement between large silk fibroin molecules, ultimately enhancing material disassembly upon exposure to water. The intermolecular and intramolecular interaction of hydrophobic amino acid domains may also be weakened. To further explore this mechanism, we have conducted several investigations of silk fibroin-Cs interactions both in water suspension and in solid, monolithic materials (i.e. film format).

In aqueous suspension, Cs does not show noticeable influence on silk nanomicelle size and on the secondary structure of the protein, as supported by DLS and CD measurements (Figure S1 and Figure 2b, respectively). Additionally, SDS-PAGE analysis of Cs-silk fibroin blends shows no aggregation or dimerization of Cs exposed to silk fibroin (Figure S1b). Investigation of the Cs-silk fibroin blends in the solid format was conducted using WAXS, SAXS, TGA, DSC, ATR-FTIR and Raman. WAXS and SAXS showed no difference between silk fibroin

and $Cs_{20}SF_{80}$ samples since the materials are not crystal dominant. Given the low impact of this study to the manuscript we did not incorporate the results of crystallography analysis in SI.

ATR-FTIR spectra of silk fibroin mixed with various content of Cs from 0% up to 40% were collected and showed no significant difference (Figure S2); all the spectra depicted a wide peak centered around 1645 cm^{-1}, corresponding to random coil. Self-deconvolution and peak fitting were carried out for all the spectra collected to quantify the secondary structure content in each sample. Incorporation of increasing concentrations of Cs in the blends did not result in a change of beta sheet content, showing that Cs did not drive a random coil to beta-sheet transition during silk fibroin assembly. Turns increased slightly as the Cs content increases, which may be attribute to the intrinsic properties of Cs, which serves as hydrophilic linkers. To further investigate the interactions between silk fibroin and Cs in solid state, Raman spectra were collected for Cs, silk fibroin, and $Cs_{20}SF_{80}$ before (solid line) and after (dotted line) methanol treatment (Figure S3). In particular, in this study we focused on the Amide I and III shifts and on the Fermi doublet peaks of the tyrosyl phenolic ring at 853 and 829 cm^{-1}. [1] In all the samples analyzed, analysis of the Amide bands showed that exposure to methanol resulted a random coil to beta-sheet transition of the silk materials, indicating Cs does not hinder polymorphic changes of the structural protein. The intensity ratio I_{853}/I_{829} has been used to study the hydrogen bonding formed by the tyrosyl phenolic-OH – a more hydrophobic tyrosine environment (i.e., reduction of structural water in the protein and of hydrogen bonding) corresponds to higher I_{853}/I_{829} ratio. As shown in Table S1, the inclusion of Cs in silk fibroin materials results in an increased I_{853}/I_{829} ratio, which corroborates the proposed mechanism that Cs reduces the formation of intermolecular hydrogen bonds.

Thermal analysis (Figure S4) showed decomposition at about 180°C for Cs, 225°C for silk fibroin and 205°C for $Cs_{20}SF_{80}$. Calorimetric analysis depicted a T_g for Cs at 60°C, for silk fibroin at 77°C and at 75°C for $Cs_{20}SF_{80.}$ In literature, this is referred to as the first T_g, i.e. $T_g(1)$ of water-containing silk materials and corresponds to the removal of free water molecules

entrapped between silk fibroin molecules during the random coil to beta sheet transition of the material. An exothermic peak was depicted at 125°C for silk fibroin only, followed by a large endothermic process. The exothermic peak is described in literature as formation of more stable structures in silk where water is present and acts as a plasticizer. The endothermic process is present in SF and $Cs_{20}SF_{80}$ samples and it corresponds to the release of some of the bound water molecules as free water and subsequent evaporation. The lack of the exothermic peak in the $Cs_{20}SF_{80}$ blend may be used as an evidence that Cs weakens the entanglement of silk fibroin molecules and reduces the formation of new, stable conformations between adjacent silk molecules upon water release. Both silk fibroin and $Cs_{20}SF_{80}$ blend showed an exothermic peak at 222°C and 214°C, respectively, which corresponds to a non-isothermal crystallization peak of silk material.[2]

Payload release profiles from SF and $Cs_{20}SF_{80}$

Payload release profiles in silk fibroin constructs have been studied extensively in controlled drug release applications,[3, 4] with most studies indicating that diffusion, swelling, and proteolytic degradation are primary drivers in this process. As targeted plant tissues are not protease-rich, we used simulated sap to investigate payload release profile. Rhodamine 6G, azoalbumin, and GFP-expressing *Rhizobium tropici* CIAT 899 (GFP-CIAT 899) were used as representative models for small molecules, large proteins, and bacteria, and their release profiles in SF and $Cs_{20}SF_{80}$ were investigated. GFP-CIAT 899 was used in the release study in lieu of *Agrobacterium* as several attempts of staining *Agrobacterium* were inconclusive due to interaction between silk fibroin and the dyes used for live/dead assays. Silk fibroin and $Cs_{20}SF_{80}$ were found to have negligible effects on fluorescence and absorbance signal. The release profile of all three payloads for both silk fibroin and $Cs_{20}SF_{80}$ follow a power law (**Figure S7**a) described by the semi-empirical model developed by Ritger and Peppas,[4]

$$f_t = \frac{M_t}{M_\infty} = kt^n, \qquad \text{(S1)}$$

which can be rewritten as $\lg(f_t) = lg(k) + nlg(t)$, where f_t is the fraction of released payload at time *t*, M_t is the amount of released payload over time *t* (unit: hour), M_∞ is the amount of released payload at infinity time, (i.e., the total payloads loaded), *k* denotes the release velocity constant determined by the structural and geometric characteristic of the system, and *n* denotes the exponent of release indicating the release mechanism. Parameters for the power law were obtained by linear fitting, shown in Table S2. Figure S7b depicts film surfaces of $Cs_{20}SF_{80}$ samples before release (silk fibroin samples have similar surfaces). Surface erosion is observed for all three releases from silk fibroin (Figure S7c), while much faster payloads release and combination of surface and bulk erosion is observed for release from $Cs_{20}SF_{80}$ (Figure S7d). Rhodamine 6G release from SF (*n* = 0.93) is *anomalous* and dominated by both diffusion and swelling. Azoalbumin release (*n* = 1.13) indicates a *Super Case II* release mechanism, possibly resulting from the secondary structure of azoalbumin (primarily α-helices) that lowers the interaction among silk fibroin chains and facilitates the disaggregation of swollen silk fibroin samples. GFP-CIAT 899 release is nearly identical to azoalbumin, but the sample surface shows protrusions, which display similar morphology to GFP-CIAT 899. All three payloads loaded into $Cs_{20}SF_{80}$ possessed a *Super Case II* release mechanism (*n*>1). This is likely due to the hydrophilicity of Cs, which dissolves easily in simulated sap and expedites the rate of sample degradation. These results show that $Cs_{20}SF_{80}$ allows for faster payload release profiles than SF, from small molecules, to large proteins, and to bacteria.

Release and transport model in xylem

The velocity of xylem sap flow is at the order of 10^{-3} m s^{-1} although it varies a lot according to the condition of measured plants during the day[5]. However, the velocity we got here is at the order of 10^{-5} to 10^{-4} m s^{-1}, which may due to the influence of injection. This gives a Péclet

number $Pe = Lu/D \sim 10$, where L is the diameter of xylem (~10^{-4} m), u is the velocity of sap flow in xylem, and D denotes the diffusion coefficient of the payload delivered in xylem sap (10^{-10} m^2 s^{-1}). Thus both advection and diffusion should be taken into consideration in this scenario. The common form of the advection-diffusion equation for an impressible fluid without source and sink is

$$\frac{\partial c}{\partial t} = \nabla \cdot (D\nabla c) - \boldsymbol{v} \cdot \nabla c \qquad \text{(S2)}$$

Since we focus on the longitudinal transport along xylem, Equation S1 can be simplified to one dimensional (1D) condition as

$$\frac{\partial c}{\partial t} = D\frac{\partial^2 c}{\partial x^2} - u\frac{\partial c}{\partial x} \qquad \text{(S3)}$$

The initial condition (IC) and boundary conditions (BCs) are as follow

IC: $c(x, 0) = 0$

BCs: $c(0, t) = c_0(t)$, $c(\infty, 0) = 0$.

Once a phytoinjector is injected into xylem, the payload is released following the power law, contributing to the concentration change at x=0 at time t $c_0(t)$ (Schematic S1). Mass conservation, i.e. payload released equals to that in the xylem, can be used to determine $c_0(t)$.

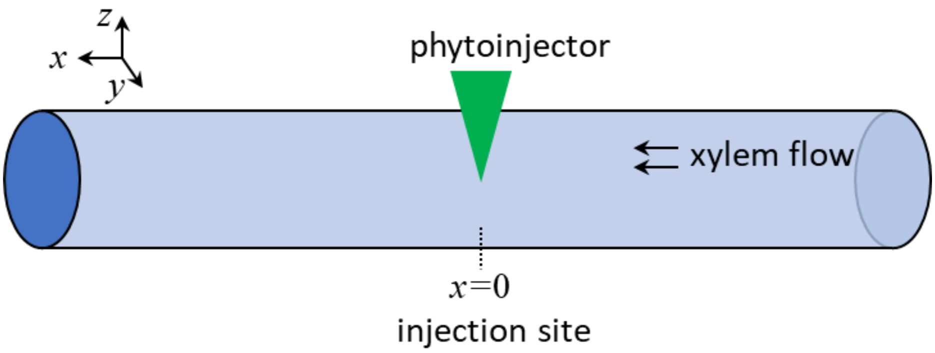

Schematic S1. Schematic of the model.

To solve this problem, let

$$c(x, t) = \Gamma(x, t)e^{\frac{ux}{2D} - \frac{u^2 t}{4D}} \qquad \text{(S4)}$$

the Equation S3 can be rewritten as

$$\frac{\partial\Gamma}{\partial t}=D\frac{\partial^2\Gamma}{\partial x^2} \tag{S5}$$

IC: $\Gamma(x,0)=0$

BCs: $\Gamma(0,t)=f(t)=c_0(t)e^{\frac{u^2t}{4D}}$, $\Gamma(+\infty,0)=0$.

Considering the Laplace transform of a function $f(x,t)$,

$$\bar{f}(x,s)=\mathcal{L}[f(x,t)]=\int_0^{+\infty}e^{-st}f(x,t)dt. \tag{S6}$$

The Laplace transform of Equation S5 is

$$\frac{d^2\bar{\Gamma}(x,s)}{dx^2}-\frac{s}{D}\bar{\Gamma}(x,s)=0 \tag{S7}$$

subjecting to boundary conditions

$\bar{f}(s)=\bar{\Gamma}(0,s)=\int_0^{+\infty}c_0(t)e^{\frac{u^2t}{4D}-st}dt$, and $\bar{\Gamma}(+\infty,s)=0$.

The solution of Equation S7 is

$$\bar{\Gamma}(x,s)=\bar{f}(s)e^{-x\sqrt{\frac{s}{D}}}=\mathcal{L}[f(t)*g(x,t)] \tag{S8}$$

which can be considered as the Laplace transform of the convolution of two functions $f(t)$ and $g(x,t)$, where $g(x,t)=\mathcal{L}^{-1}\left[e^{-\frac{x}{\sqrt{D}}\sqrt{s}}\right]=\frac{x}{\sqrt{4\pi Dt^3}}e^{-\frac{x^2}{4Dt}}$.

The inversion of $\bar{\Gamma}(x,s)$ gives

$$\Gamma(x,t)=\int_0^t f(\tau)g(x,t-\tau)d\tau=\frac{x}{\sqrt{4\pi D}}\int_0^t\frac{c_0(\tau)}{\sqrt{(t-\tau)^3}}e^{\frac{u^2\tau}{4D}-\frac{x^2}{4D(t-\tau)}}d\tau. \tag{S9}$$

The concentration thus is

$$c(x,t)=\frac{x}{\sqrt{4\pi D}}e^{\frac{ux}{2D}-\frac{u^2t}{4D}}\int_0^t\frac{c_0(\tau)}{\sqrt{(t-\tau)^3}}e^{\frac{u^2\tau}{4D}-\frac{x^2}{4D(t-\tau)}}d\tau,\ (x>0) \tag{S10}$$

$$c(x,t)=\frac{-x}{\sqrt{4\pi D}}e^{\frac{ux}{2D}-\frac{u^2t}{4D}}\int_0^t\frac{c_0(\tau)}{\sqrt{(t-\tau)^3}}e^{\frac{u^2\tau}{4D}-\frac{x^2}{4D(t-\tau)}}d\tau,\ (x<0) \tag{S11}$$

Thus the concentration for the whole field is

$$c(x,t)=\frac{|x|}{\sqrt{4\pi D}}e^{\frac{ux}{2D}-\frac{u^2t}{4D}}\int_0^t\frac{c_0(\tau)}{\sqrt{(t-\tau)^3}}e^{\frac{u^2\tau}{4D}-\frac{x^2}{4D(t-\tau)}}d\tau. \tag{S12}$$

In addition, the concentration must meet mass conservation

$$M_t = M_\infty k t^n = \int_{-\infty}^{+\infty} c(x,t)dx. \quad \text{(S13)}$$

This integral equation determines boundary condition $c(0,t) = c_0(t)$ and thus $c(x,t)$. While it is hard to explicitly solve the integral equation, we can solve it numerically. By Taylor series, we have

$$\left(\frac{\partial c}{\partial t}\right)_i^n = \frac{c_i^{n+1} - c_i^n}{\Delta t} + O(\Delta t),$$

$$\left(\frac{\partial c}{\partial x}\right)_i^n = \frac{c_{i+1}^n - c_{i-1}^n}{2\Delta x} + O(\Delta x^2)$$

$$\left(\frac{\partial^2 c}{\partial x^2}\right)_i^n = \frac{c_{i+1}^n - 2c_i^n + c_{i-1}^n}{\Delta x^2} + O(\Delta x^2)$$

Where *n* denotes time *t* and *i* is position *x*.

Equation S3 can be approximated as

$$\frac{c_i^{n+1} - c_i^n}{\Delta t} = D\frac{c_{i+1}^n - 2c_i^n + c_{i-1}^n}{\Delta x^2} - u\frac{c_{i+1}^n - c_{i-1}^n}{2\Delta x} + O(\Delta t, \Delta x^2) \quad \text{(S14)}$$

and

$$c_i^{n+1} = c_i^n - \frac{u\Delta t}{2\Delta x}(c_{i+1}^n - c_{i-1}^n) + \frac{D\Delta t}{\Delta x^2}(c_{i+1}^n - 2c_i^n + c_{i-1}^n) \quad \text{(S15)}$$

The code was written in MATLAB R2019a. Parameters to carry out the simulation used are $D = 4 \times 10^{-10} m^2/s$, $u = 5 \times 10^{-5} m/s$, $k = 0.038$ (for time unit minute), and $n = 1.61$. The power law release describes well the first 60% payload release but not for 100%. Thus, our model well describes the release and transport in the first 5 minutes only. For longer time period, the payload loaded to other parts of the phytoinjector may also be released and contributes as payload source at the injection site, which invalidates the mass conservation assumption used here.

Lucas-Washburn model for phytosampler

Reswelling of the phytoinjectors and diffusion of metabolite and catabolite in silk phytosampler was modeled with a Lucas-Washburn equation.[6] The fitting was carried out in MATLAB

R2019a Curve Fitting Toolbox on collected data of penetration depth of water frontier in a phytosampler over time.

The fitting equation is

$$H = 36.42\sqrt{t - 54.32}, \quad \text{(S16)}$$

where H is the penetration depth, t is time (unit second). The adjusted R^2=0.9932. The time t_0=54.32 s may attribute to the cone shape of the phytosampler, which does not match the 1D case for Lucas-Washburn model.

Estimation of the amounts of payloads delivered by phytoinjectors

We have estimated the amount of cargo molecules delivered for a payload equivalent to 10wt% and compared it with the functional amount found in several plant tissues. In particular, we found that the deliverable weight of cargo molecules is in the order of 10s of ng per phytoinjector.

The total volume of xylem and phloem phytoinjector (V_{phyt}) is 18.74±1.05 nl and 9.11±1.83 nl, respectively. Given that Cs-silk fibroin blends have a density of 1.40 g cm^{-3} (which is equal to 1.4 µg nl^{-1}), the weight of xylem and phloem phytoinjector is 26.24 µg and 12.75 µg, respectively. Let's define that the phytoinjector tip volume of V_{tip}=3% of V_{phyt} (the tip length 100 - 200 µm). Assuming to load the phytoinjector with a 10wt% payload, this would correspond to the loading of 78.7 ng of cargo molecules for xylem phytoinjector and of 38.3 ng of cargo molecules for phloem phytoinjector (Table S3).

Plant hormones level is usually in the range of 0.1-50 ng g^{-1} of fresh weight.[7] As hormones are found in specific tissues such as shoot apical meristem and leaves, which have a weight in the order of tens to hundreds of milligrams, the delivered level of hormones by phytoinjectors would provide the plant with physiologically relevant quantities of hormones.

Micronutrients are present in plant tissues at concentration of ppm per dry weight, which approximately equals to 100ng g^{-1} fresh weight. This makes phytoinjectors suitable for

delivering a wide range of micronutrients, including Cu, Mo, and Ni. (Table S4 [8]). Note that micronutrients deficiency does not mean we need to deliver adequate concentration of micronutrients to plants. In addition, according to our experience, less than 1 ng of siRNA per leave of *Nicotiana benthamiana* result in the suppression of chlorophyll synthesis, indicating a very low functioning quantity of iRNA.

Experimental Section

Extraction of silk fibroin: The aqueous silk fibroin solution was prepared from *Bombyx mori* cocoons as described with modification.[9] Briefly, dime size cocoon pieces were boiled for 45 minutes to remove sericin in 0.02 M sodium carbonate solution and dried overnight after thorough rinse in MilliQ water. The dried silk fibroin fibers were then dissolved in 9.3 M lithium bromide solution at 60 ˚C for 4 h, followed by dialysis against MilliQ water in a Slide-a-Lyzer dialysis cassette (MWCO 3500, Pierce, Rockford, IL) for 48 h. After centrifuge, the supernatant was obtained and stored at 4 ˚C prior to use. The final concentration of silk fibroin is roughly 7% w/v, determined by weighing the residual of 1 mL solution.

Cs preparation: Cs was prepared following the method described previously with modification.[10] Alpha-chymotrypsin was added to aqueous silk fibroin solution by an enzyme to substrate weight ratio 1:100, followed by incubation at 37 ˚C for 24 h. The gel formed was then centrifuged at 4800 ×g for 30 minutes. The supernatant (Cs) was collected and kept at 80 ˚C for 20 minutes to denature alpha-chymotrypsin. The solution was centrifuged again, and the supernatant was stored at 4 ˚C prior to use. The concentration was determined by weighing dry residual.

Gel electrophoresis: The electrophoretic mobility of silk fibroin, Cs, and $Cs_{20}SF_{80}$ were determined using sodium dodecyl sulfate polyacrylamide gel electrophoresis (SDS-PAGE). 100 µg silk fibroin, 300 µg Cs, and 100 µg $Cs_{20}SF_{80}$ were mixed with 2X Laemmli Sample Buffer and loaded into a precast 4-15% polyacrylamide gel (Bio-Rad Laboratories, Hercules, CA). The gel was run for 23 minutes at 200 V with a prestained recombinant protein mixture as reference (Bio-Rad Laboratories). The gel was first washed twice with 5% (v/v) methanol in MilliQ water for 15 min each time. The gel was then stained by 0.001% crystal violet with 10% (v/v) methanol and 1.5% (v/v) acetic acid overnight.

Dynamic light scattering (DLS): Zeta Potential Analyzer (Brookhaven Instruments Corp., Holtsville, NY) was used to measure the particle size in resuspended solution at a concentration of 1 mg ml^{-1} dry material. Each measurement was 180 s and at least three measurements were carried out per sample's type.

Circular dichroism (CD): CD experiments were conducted with a JASCO Model J-1500 Circular Dichroism Spectrometer (JASCO Co., Japan). Aqueous solutions were diluted to 0.01% w/v, loaded into a 1 mm path quartz cell (Starna Cells, Inc., Atascadero, CA), and scanned at 25 °C with a resolution of 0.5 nm and a 4 s accumulation time at the rate of 50 nm min^{-1} from 250 nm to 185 nm wavelength. The results were averaged from three measurements.

Fourier Transform Infrared Spectroscopy (FTIR): IR measurements were carried out on a Spectrum 65 (PerkinElmer, Waltham, MA) equipped with an attenuated total reflection (ATR) generic UATR crystal, with a resolution of 4 cm^{-1} and accumulation of 32 scans from 4000 and 650 cm^{-1}. Films were cast on PDMS, dried overnight, and kept in a desiccator for 24 h to remove surface water. Analysis was performed based on the Amide I region (1595−1705 cm^{-1}) by OriginPro 2017 software (OriginLab Corporation, Northampton, MA), following the previously described method.[11]

Raman spectroscopy: Raman spectra were obtained with a Renishaw inVia Raman Microscope (Renishaw PLC, Wotton-under-Edge, United Kingdom) with a laser 785 nm and a 10X objective. Data were collected and analyzed with software WiRE v5.2. Cs, silk fibroin, and $Cs_{20}SF_{80}$ were cast on PDMS and dried in a fume hood overnight. SF and $Cs_{20}SF_{80}$ films were immersed in 80% v/v methanol for 5 minutes described as 'methanol treatment' in the main text. Cs samples did not form a film but fragments, which were immersed into 1 ml 80% v/v methanol in a 6 mm petri dish until the completion of evaporation of liquid. H_2O_2 solution was mixed with $Cs_{20}SF_{80}$ solution at a material ratio of 5:1 and dried overnight in a hood. Three samples were tested for each case.

Thermogravimetric analysis (TGA): TGA curves were collected via a Discovery TGA model (TA instruments, New Castle, DE). Specimens were heated up at a rate of 10 °C min^{-1} from 40 °C to 500 °C in nitrogen with a rate of 25.0 ml min^{-1}. Three samples were tested for each case.

Differential scanning calorimetry (DSC): DSC curves were collected via a Discovery DSC model (TA instruments, New Castle, DE). Specimens were heated up at a rate of 10 °C min^{-1} from 40 °C to 230 °C (Cs) or 270 °C (SF and $Cs_{20}SF_{80}$). Data were replotted with mass loss taken into consideration according to TGA results. Three samples were tested for each case.

Preservation of hydrogen peroxide and HRP: H_2O_2 can be enzymatically degraded by HRP, the product of which oxidizes 3,3',5,5'-Tetramethylbenzidine (TMB) and generates a deep blue color. Upon addition of acid solution, the blue color turns to yellow that can be recorded absorbance at 450 nm. Briefly, for hydrogen peroxide preservation, H_2O_2 was added to CsSF blend solution, with a final H_2O_2 concentration 0.1% w/v and CsSF material concentration 6% w/v. Films were prepared by dropping 50 µl solution on PDMS and drying overnight in a fume hood. Each film was dissolved in 500 µl water for absorbance reading. 5 µl of the sample solution was mixed with 80 µl of TMB solution and incubated for 1 minute at room temperature before the addition of 100 µl 0.1 M sulfuric acid. Absorbance was detected at 450 nm with reference at 620 nm by a Tecan microplate reader (Tecan Group Ltd, Switzerland). HRP preservation shared a similar protocol with the modification where HRP was added to CsSF blend solution to prepare films. The standard curve is in Figure S11.

Bacteria culture: *Rhizobium tropici* CIAT899 expressing bacterial GFP was obtained from Miguel Lara.[12] R. tropici was cultured at 30 °C to OD600 of 1 following the instructions before use. GFP gene was cloned into pEAQ-HT vector and transformed into *A. tumefaciens* strain (LBA4404). Transformants were cultivated and selected at 30 °C for 24-36 h to OD600 of 1.5 in YM medium (0.4 g L^{-1} yeast extract, 10 g L^{-1} mannitol, 0.1 g L^{-1} NaCl 0.2 g L^{-1} $MgSO_4 \cdot 7H_2O$,

0.5 g L^{-1} $K_2HPO_4 \cdot 3H_2O$, 15 g L^{-1} agar, pH 7) supplemented with 50 μg mL^{-1} rifampicin, 50 μg mL^{-1} kanamycin, and 50 μg mL^{-1} streptomycin.

Preservation of Agrobacterium tumefaciens: *A. tumefaciens* was cultured to OD600 1, centrifuged down at 3000 × g for 30 minutes and resuspended by SF and $Cs_{20}SF_{80}$ to the same volume. Films were prepared by dropping 50 μl suspension on PDMS and drying overnight in a fume hood. The films were dissolved in 0.9% sterile NaCl solution and then spread on an agar plate for colony counting. A series of dilutions were prepared for better counting results.

Mechanical properties tests: Cs/silk fibroin solutions were cast on PDMS, dried overnight in a fume hood at room temperature, and cut into ribbons. Film tensile experiments were carried out on a Dynamic Mechanical Analysis (DMA) Q800 model (TA instruments, New Castle, DE) with a strain rate of 0.5% min^{-1} at room temperature. The static ultimate compression strength of phytoinjectors and puncture of plants' tissues were also conducted on a Dynamic Mechanical Analysis (DMA) Q850 model (TA instruments, New Castle, DE) using compression clamps at a loading speed of 1 mm min^{-1}. The sixth compound leaves from 7-week-old tomato plants (*Solanum lycopersicum*) having 13 compound leaves were collected to get tomato petiole and leaflet samples. Stems between the sixth and eighth leaves were collected from multiple plants as stem samples. The sixth leaves from 6-week-old tobacco (*Nicotiana benthamiana*) were collected as petiole and leaf samples. Green branches (not woody bark) and leaves of a navel orange tree (*Citrus sinensis*) were used as tissue samples. At least 3 samples were tested for each case. Nanoindentation measurements were performed on a Hysitron TriboIndenter with a nanoDMA transducer (Bruker, Billerica, MA). Samples were indented in load control mode with a peak force of 500 μN and a standard load-peak hold-unload function. Reduced modulus was calculated by fitting the unloading data (with upper and lower limits being 95% and 20%, respectively) using the Oliver-Pharr method. Each type of sample was prepared and indented in triplets to ensure good fabrication repeatability. For each sample, indentation was performed at

a total of 49 points (7×7 grid with an increment of 20 μm in both directions) to ensure the statistical reliability of the modulus measurements.

Payloads release: Simulated sap was prepared according to the xylem exudate.[13] Rhodamine 6g and azoalbumin were added to SF and $Cs_{20}SF_{80}$ (6% w/v of dry materials) to get a final concentration of 0.1 mM and 2 mg ml^{-1}, respectively. R. tropici was centrifuged at 3000 × g for 30 minutes and resuspended by SF and $Cs_{20}SF_{80}$ to get an OD600 of 1. The solutions were then cast on PDMS and dried overnight in a hood. The films were then cut into discs and attached to the bottom of a well of a 48 well plate, enabling only one side of the disc exposed to simulated sap. 1 ml of fresh simulated sap was added after the previous solution was collected for measurement. Released rhodamine 6g and GFP-expressing *R. tropici* were monitored based on fluorescence intensity (excitation at 524 nm and 499 nm, emission at 550 nm and 520 nm). Released azoalbumin was monitored based on absorbance at 410 nm. At least three samples were tested for each case. The standard curve is in Figure S11.

Master and negative mold fabrication: The aluminum master was fabricated by computer numerical control (CNC) machining with a 1/32" flat end mill for rough milling, followed by a 1/64" ball end mill for finishing. The templates were then chemically etched to the desired topologies based on application by aluminum etchant type A (Transene, Danvers, MA). To produce negative, Poly(dimethyl siloxane) (PDMS) (Sylgard 184, Dow-Corning, Midland, MI) was cast over Al master in a 60 mm petri dish, degassed, and finally incubated at 70 ˚C for 2 h.

Phytoinjector fabrication: The desired amount of payloads were mixed with $Cs_{20}SF_{80}$ solution and added to negative PDMS molds, followed by centrifuge at 1200 ×g for 15 minutes. Molds were then kept in a fume hood to dry at room temperature overnight. The phytoinjector array was then cut into smaller arrays by a razor blade for tissue application.

Plant materials: Tobacco (*Nicotiana benthamiana*) and tomato (*Solanum lycopersicum*) plants were grown in pots in a plant chamber with ambient temperature 25 ˚C day/20 ˚C night and a

10 h photoperiod. Tobacco plants between 4-6 weeks old after germination are used for experiments, while tomato plants were used when they are 5-8 weeks old from seeds. A navel orange tree (*Citrus sinensis*) was grown in a 15' pot with regular water and fertilizer feeding in 25˚C day/20˚C night and 12 h photoperiod.

Histology: Tomato plant tissues of interest were collected and kept in 10% formalin for 24 h, followed by immersion in 70% ethanol before processing by a Rapid Biopsy Processing on the Vacuum Infiltrating Tissue Processor for paraffin filling. 10 μm thick slices were prepared by a microtone and stained by Safranin O stain and Fast Green after deparaffinization.

Payloads delivery to tomato plant: 5(6)-carboxyfluorescein diacetate (1 mM) and rhodamine 6G (1 mM) was mixed with 6% wt/v $Cs_{20}SF_{80}$ solution (volume ration 1:100) to fabricate phytoinjectors which were used to demonstrate the capability of phytoinjector to deliver payloads to xylem and phloem, repectively. The upstream and downstream cross-sections along the petiole were observed under microscope to record the appearance of fluorescence due to delivery and transport of 5(6)-carboxyfluorescein diacetate and rhodamine 6G with fixed light intensity and exposure time (20 ms). The petiole was also sliced longtutionally to image distribution profile of 5(6)-carboxyfluorescein diacetate in xylem. Images were analyzed with imageJ 1.52i. Fluorescence intensity was used to represent the concentration of 5(6)-carboxyfluorescein diacetate within the range we used. Fluorescence signal was integrated along the radial direction. 50 μl 15 mg/ml D-luciferin potassium salt solution was added to 2.5 ml 6% wt/v $Cs_{20}SF_{80}$ solution to fabricate luciferin loaded xylem phytoinjectors. Luciferin-loaded phytoinjectors were injected into petioles near a termial leaflet of a tomato compound leaf. Solution containing 150 uM ATP and 5 mM $MgCl_2$ and 10 μg ml^{-1} luciferase was delivered to the leaflet via foliar infiltration. The leaflet was then imaged in a dark room via a Nikon 3400 camera with an exposure time of 30 s. Images were modified with an ad-hoc Matlab script to double the intensiy of the RGB signal for display purposes due to the original low luminescence

intensiy. Similarly, luciferin and luciferase loaded phytoinjectors were injected on petiole near a leaflet and ATP and $MgCl_2$ were delivered externally. The leaflet was imaged with exposure time 120 s and ad-hoc Matlab script was applied to double the intensiy of the RGB signal for display purposes.

Sampling via xylem phytoinjector fabricated from pure SF: luciferin and $MgCl_2$ were loaded to $Cs_{20}SF_{80}$ xylem phytoinjectors, which were then injected to tomato petioles. Phytosamplers fabricated from pure silk fibroin were injected to the nearby position on the same petiole, supposing they reach the same xylem of $Cs_{20}SF_{80}$ xylem phytoinjectors. The phytosamplers were flipped on a glass slide and a drop of ATP and luciferase solution was added to image luminiscence. The camera exposure time was set to 30 s. 1% agar gel was prepared in a petri dish with thickness of ~3 mm in order to maintain high transparency. Phytosamplers were injected into the agar gel and images were taken with a Nikon TE2000-E microscope (Nikon Inc., Minato City, Tokyo, Japan) using a 4x objective at 10 s intervals to investigate the movement of water from the gel to inside the phytosampler, thus, the sampling behavior. The movement of the interface between dry silk fibroin and rehydrated silk fibroin along the phytosampler length direction was collected via imageJ and used to plot the penetration length *vs* time.

Agrobacterium mediated gene transfer to shoot apical meristem and leaves: Agrobacterium loaded phytoinjectors were injected into SAM, young leaf, and mature leaf of 5-week-old tobacco. Fluorescent leaves were imaged via a Invitrogen Safe Imager 2.0 Blue-Light Transilluminator (Thermo Fisher Scientific Inc., Waltham, MA) and Nikon TE2000-E microscope (Nikon Inc., Minato City, Tokyo, Japan) 2 weeks post injection when the SAM grew to a leaf.

Supporting Figures

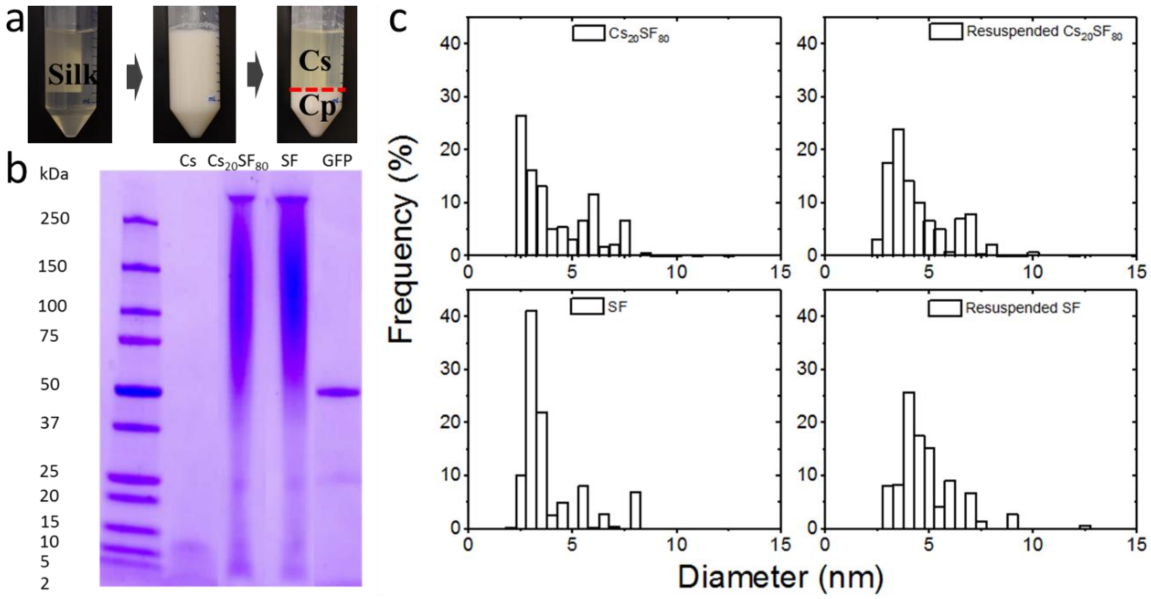

Figure S1. Cs fabrication and material size distribution. a, Photographs of: silk fibroin solution, gel formed after 24 h incubation at 37 °C of silk fibroin and alpha-chymotrypsin, and Cs after centrifuge. **b,** SDS-PAGE of silk fibroin (SF), Cs, $Cs_{20}SF_{80}$, and GST tagged GFP (~53kDa). **c,** Size distribution of as prepared $Cs_{20}SF_{80}$, SF and resuspended $Cs_{20}SF_{80}$ and SF. Pure Cs solution has a hydrodynamic radius below 1 nm.

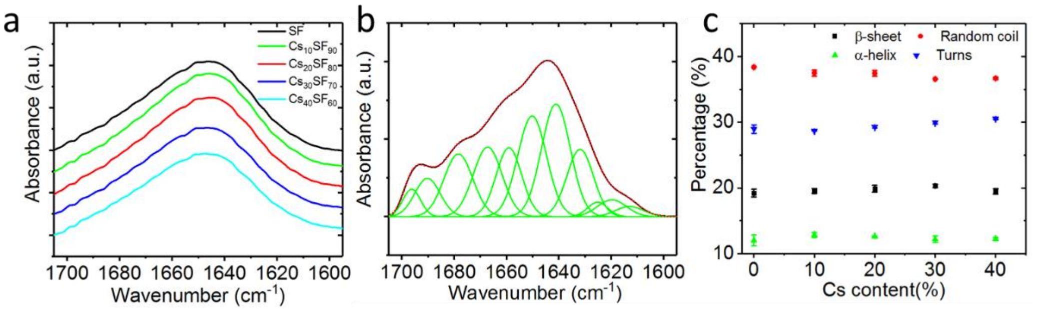

Figure S2. ATR-FTIR spectra of CsSF blend and quantification of secondary structure. a, ATR-FTIR spectra of CsSF blend with increasing Cs content. All the investigated ratios of silk:Cs showed similar spectra with a strong peak at 1645 cm^{-1} indicating water-soluble random coil conformation. **b,** Self-deconvolution curve of the ATR-FTIR spectrum of SF and peak fitting. Black solid line is the self-deconvoluted curve, red dot line is the fitted curve by individual peaks (green). **c**. Percentage of secondary structures in CsSF blends with increasing Cs content. Error bar means s.d.

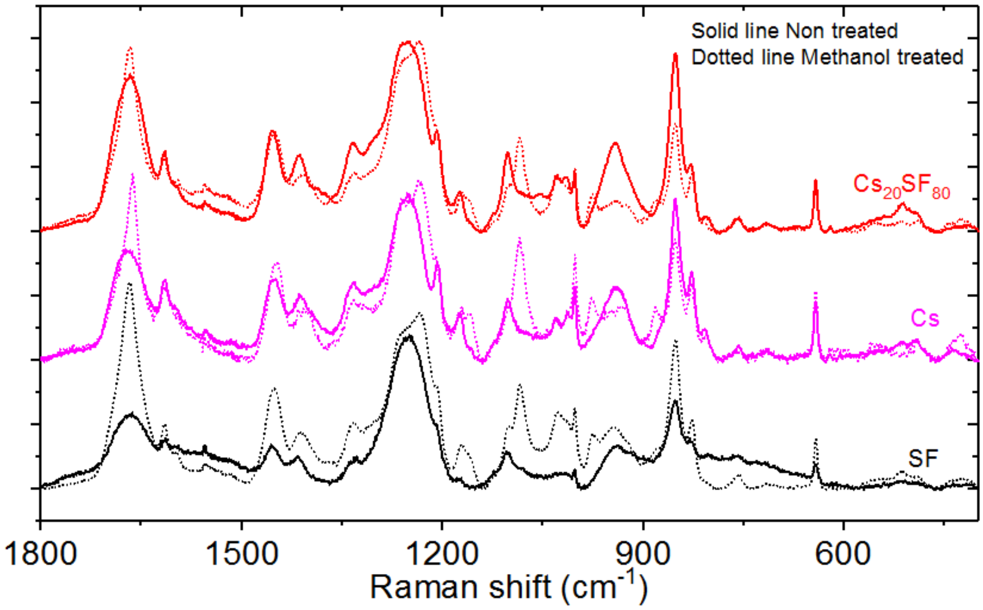

Figure S3. Raman spectra of Cs, $Cs_{20}SF_{80}$, and SF. Solid lines indicates samples that are as prepared while dotted lines refer to samples treated in 80% v/v methanol. Cs shows a polymorphic behavior upon exposure to methanol as it undergoes a random coil to beta-sheet transition (changes in Amide I and III bands).

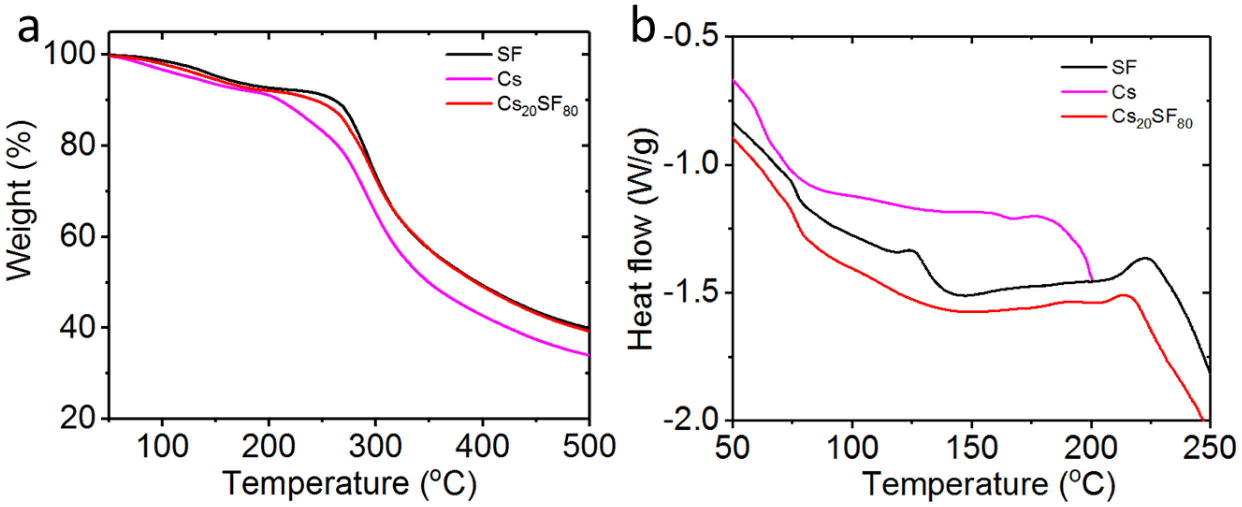

Figure S4. TGA (a) and DSC (b) thermograms of Cs, $Cs_{20}SF_{80}$, and SF.

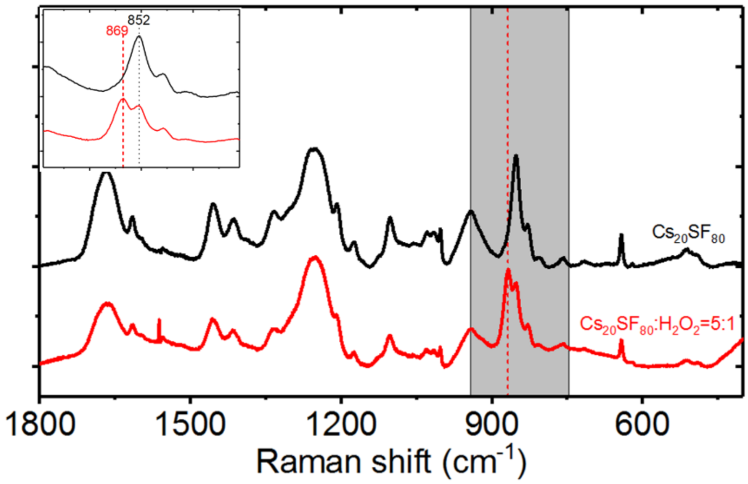

Figure S5. Raman spectra of $Cs_{20}SF_{80}$ with and without H_2O_2. The characteristic band of H_2O_2 880 cm^{-1} shifts to 869 cm^{-1} due to the contribution of a protein band at 852 cm^{-1}.

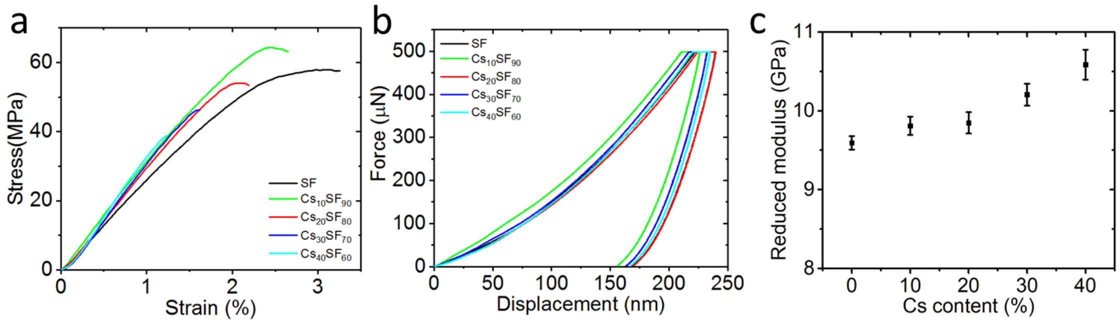

Figure S6. Mechanical properties of CsSF blend. a, Stress-strain curves of CsSF blends. **b,** Force-displacement curves of nanoindentation of CsSF blends. **c,** Reduced Young's modulus of CsSF blends. Error bar means s.d.

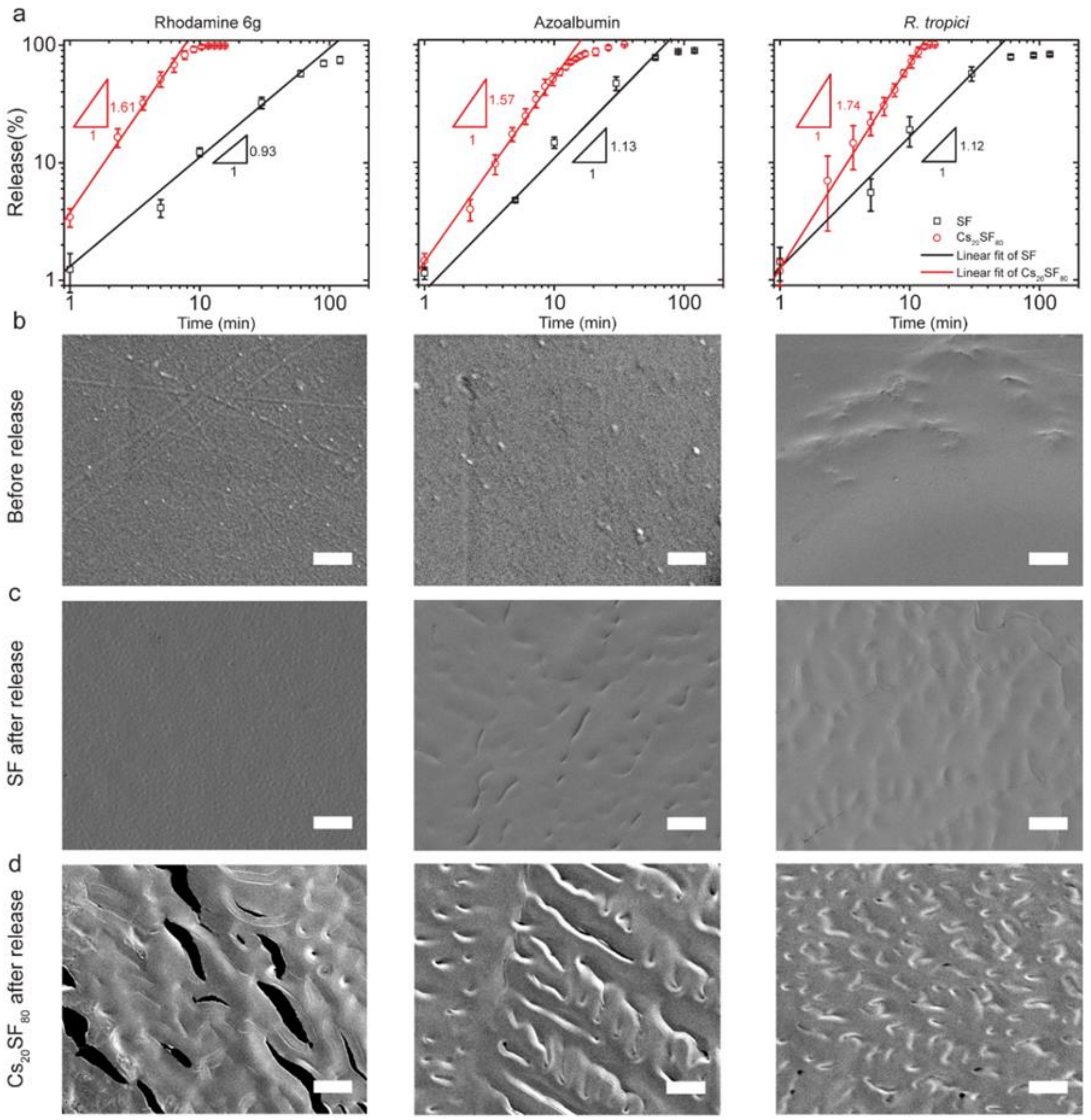

Figure S7. Release of payload models in simulated sap. a, Rhodamine 6g (left), azoalbumin (middle), and *R. tropici* (right) preserved in SF and $Cs_{20}SF_{80}$ release in simulated sap. All the payloads encapsulated in the two materials follow a power law release. $Cs_{20}SF_{80}$ showed an increased release rate than SF. **b,** Scanning electron micrographs of $Cs_{20}SF_{80}$ with different payloads. The surfaces of rhodamine 6g and azoalbumin loaded are flat and smooth, while the surface of *R. tropici* loaded materials shows the bacteria profiles. **c** and **d,** SEM images of SF and $Cs_{20}SF_{80}$ materials after 5 mins exposure to simulated sap. Scale bar, 10 µm. Data are mean ± s.d (n=3).

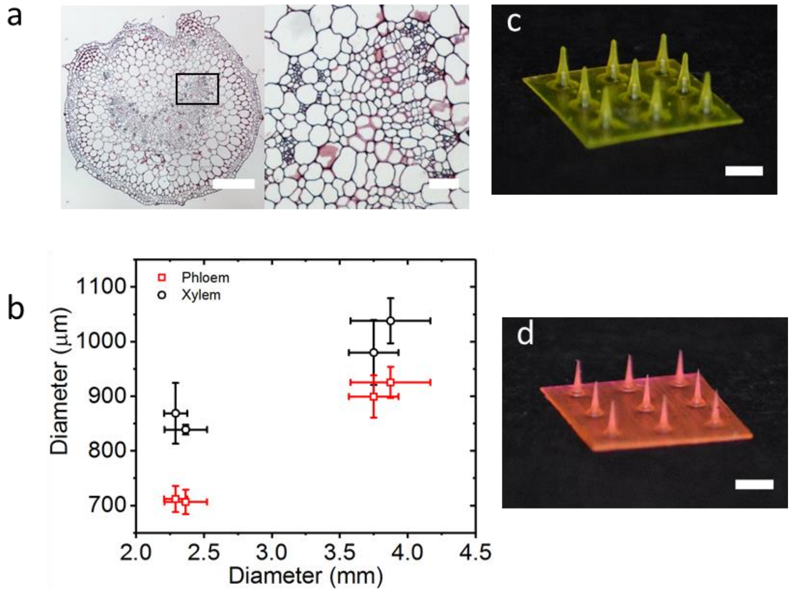

Figure S8. Phytoinjectors targeting on xylem and phloem of tomato plants. **a**, Tomato petiole cross section. Phloem (deep green) and xylem (pink) are regularly arranged. Scale bar 500 μm for the left and 50 μm for the right. **b**, Depth of phloem and xylem in tomato petiole. **c** and **d**, photograph of phytoinjectors for xylem and phloem, respectively. Scale bar 1 mm. Error bar means s.d.

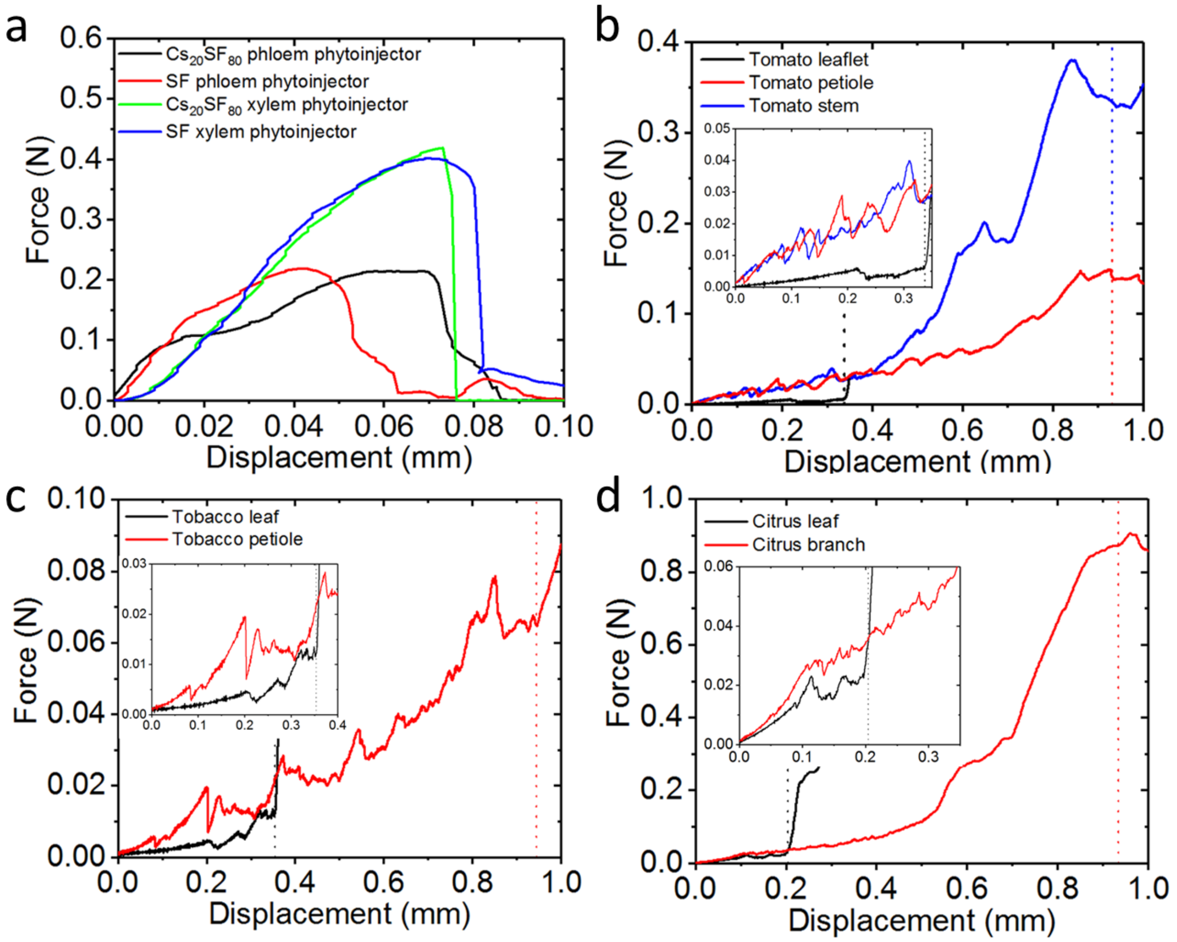

Figure S9. Mechanical behavior of phytoinjectors and plant tissues during injection with a xylem phytoinjector. a, Mechanical behavior of xylem and phloem phytoinjectors fabricated from $Cs_{20}SF_{80}$ and SF under compression. The phytoinjectors mainly break due to bending because the inevitable lateral force exerted during compression. The phloem phytoinjector fabricated from $Cs_{20}SF_{80}$ may undergo material cracking as the force was maintained around 0.1 N where the displacement is from 15 µm to 25 µm (The tip of a phloem injector is < 10 µm in diameter). Reaction forces during injection of a xylem phytoinjector into tomato (**b**), tobacco (**c**), and citrus (**d**). Dotted lines represent the completion of the injection, where the whole phytoinjector was inside the tissue plant or the tissue (leaf) was injected through.

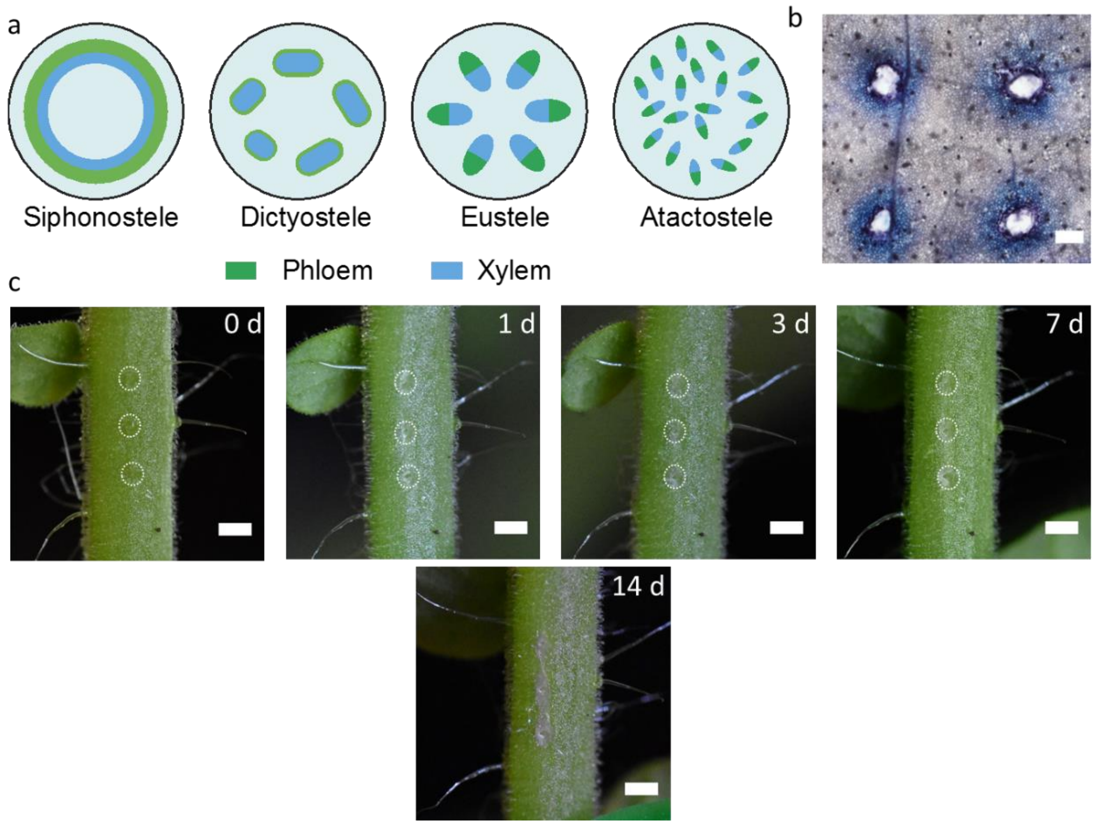

Figure S10. Stele types and wound caused by phytoinjectors a, Different types of steles. **b,** Leaf cell viability post injection. Cells stained blue by toluidine blue are dead while not stained are alive. Scale bar 100 µm. **c,** Wound on tomato petiole caused by xylem phytoinjectors, immediate, 1, 3, 7, and 14 days post injection. Scale bar 1 mm.

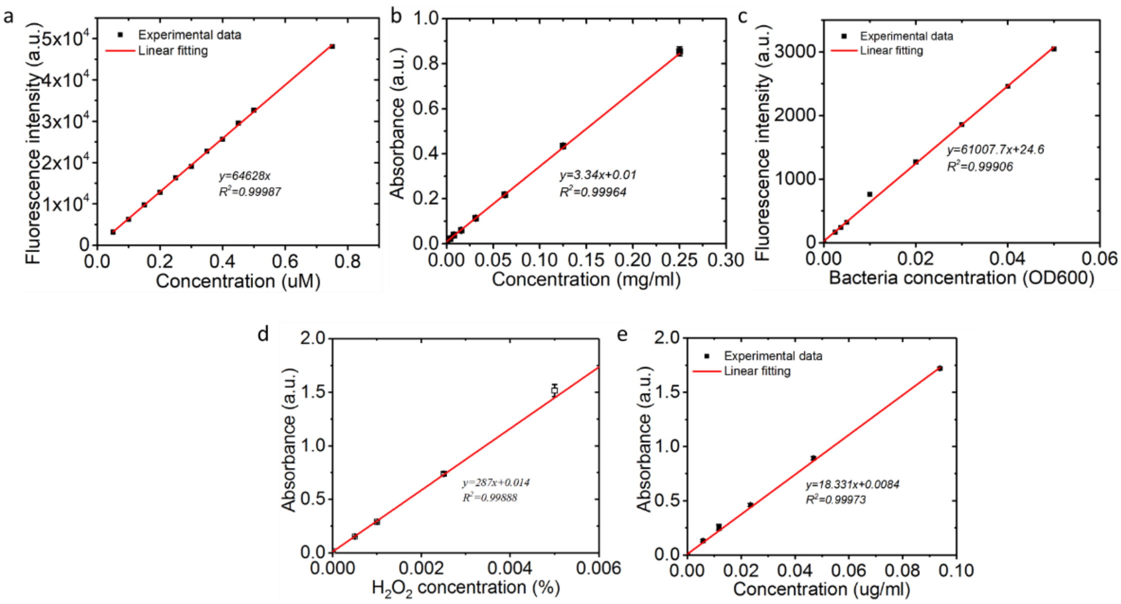

Figure S11. Standard curves. **a**. rhodamine 6G, **b**. azozlbumin, **c**. *R. tropici*, **d**. H_2O_2, and **e**. HRP, respectively. R^2 is adjusted R-squared. Error bar means s.d.

Supporting Tables

Table S1. I_{853}/I_{829} ratio

I_{853}/I_{829}	Silk fibroin	Cs	$Cs_{20}SF_{80}$
Non treated	1.69	1.94	2.47
Methanol treated	2.26	2.84	1.94

Table S2. Power law fitting parameters of payloads release. Data are mean ± s.d.

Material	SF			$Cs_{20}SF_{80}$		
	k[a)]	n	R^2	k[a)]	n	R^2
Rhodamine 6G	0.59±0.04	0.93±0.06	0.9823	27.54±5.51	1.61±0.07	0.9926
Azoalbumin	0.82±0.34	1.13±0.16	0.9395	8.63±0.72	1.57±0.04	0.9957
R. tropici	1.22±0.17	1.12±0.08	0.9850	15.52±1.22	1.74±0.04	0.9973

[a)]The unit for time *t* is hour for paramater k.

Table S3. Estimation of the amount of payload delivered by a phytoinjector.

	Volume (nl)	Weight (µg)[a*]	Tip weight (µg)	Payload weight (ng)[b*]
Xylem phytoinjector	18.74±1.05	26.236	0.787	78.7
Phloem phytoinjector	9.11±1.83	12.754	0.383	38.3

[a*] The density of 1.40 g cm^{-3} is used to do calculation.
[b*]The payload weight is supposed to be 10% of the phytoinjector.

Table S4. Micronutrients concentration in plant.[8]

Element	Range of Concentrations (ppm)	Adequate Concentration (ppm)	Adequate Concentration (ng per gram fresh weight)
Cu	2–50	6	600
Mo	0.01–10	0.1	10
Ni	0.01–5	0.05	5

MATLAB code for payloads release

```
function release
%This function is used to solve the release of payloads from
%phytoinjector and transport in xylem and phloem.
%The model is 1D advection-diffusion equation.

%Constants
%D=7.0*10^(-10);  %Diffusion coefficient of Mg2+ ion in water
%D=4.0*10^(-10);  %Diffusion coefficient of R6G/5(6)-Carboxyfluorescein diacetate in
water
%D=6.1*10^(-11);  %Diffusion coefficient of albumin in water
%u=10^(-3);       %Velocity of sap in xylem
%u=10^(-4);       %Velocity of sap in phloem

D=4.0*10^(-10);
u=5*10^(-5);
k=0.038;         %M=Minf*k*t^(nn), M/Minf<=60, tmax is calculated
nn=1.61;
Minf=1;

tmax=round(60*(0.6/k)^(1/nn));%Total time,unit second
%tmax=300;
dt=0.001;            %Time step,
tN=tmax/dt;

L=0.1;         %2N+1 is the number of points along x L=0.1m
N=10000;
dx=L/(2*N);

%Matrice
c_tn=zeros(1,2*N+1); %t=n*dt Concentration of payloads at each point
c_tn1=zeros(1,2*N+1); %t=(n+1)*dt Concentration of payloads at each point
c_x0=zeros(1,tN+1);  %c_x0(t), Concentration at x=0, c(N+1). t=0,c0(1)=0
cinf=0;          %Concentration at infinite, c(1)=c(2*N+1)=0

x=-L/2:dx:L/2;

t_output=[60 180 300];   %used to determine when to write c(x,t), -L/2<=x<=L/2
ct=zeros(length(t_output),2*N+1);
ij=0;

for n=0:tN-1
   t=(n+1)*dt;
   cn_tem=c_tn;

   m_err=1e-6;
   aa_lower=0;aa_upper=1;aa=1;
   while abs(m_err)>1e-8
      if aa>0
         [aa,aa_upper,aa_lower]=increase(m_err,aa,aa_upper,aa_lower);
```

```
        %else
         %   aa_lower=-1;aa_upper=0;aa=-1;
         %   [aa,aa_upper,aa_lower]=increase(m_err,aa,aa_upper,aa_lower);
        end

        %Initialization
        M_tn=Minf*k*(dt/60)^nn*((n+1)^nn-(n)^nn); % material released at tn
        c_x0(n+2)=c_x0(n+1)+aa*M_tn/dx;  %c0(x=0,t)
        cn_tem(N+1)=c_x0(n+2);

        c_tn1(1)=cinf;       %BCs x=-L/2
        c_tn1(2*N+1)=cinf;   %x=L/2
        for i=2:N*2
            c_tn1(i)=cn_tem(i)-...
                u*dt/(2*dx)*(cn_tem(i+1)-cn_tem(i-1))+...
                D*dt/(dx)^2*(cn_tem(i+1)-2*cn_tem(i)+cn_tem(i-1));
            if c_tn1(i)<0
                c_tn1(i)=0;
            end
        end
        %material released error during n to n+1 dt period
        m_err=sum((c_tn1-c_tn))*dx-M_tn;
    end

    c_x0(n+2)=c_tn1(N+1);  %c(x=0,t=t)
    c_tn=c_tn1;

    %used to determine when to write c(x,t), at every 0.1*tmax
    if ismember((n+1)*dt,t_output)
        ij=ij+1;
        ct(ij,:)=c_tn;
    end

end


t=0:dt:tmax;
fileID = fopen('concentration vs time.txt','w');
fprintf(fileID,'%10s %12s %12s %12s\r\n','x','t0','t1','t2');
fprintf(fileID,'%10.8f %12.8f %12.8f %12.8f\r\n',[x;ct]);
fclose(fileID);

fileID2 = fopen('c_x0 vs time','w');
fprintf(fileID2,'%10s %12s\r\n','time(s)','c_x0');
fprintf(fileID2,'%10.8f %12.8f\r\n',[t;c_x0]);
fclose(fileID2);

figure
ax1=subplot(2,1,1);
grid on
plot(ax1,t,c_x0)
```

```
title('concentration at x=0 vs. time')
xlabel(ax1,'Time(s)')
ylabel(ax1,'Concentration')

ax2=subplot(2,1,2);
grid on
plot(ax2,1000*x,ct(:,:))
title('concentration distribution at different time')
xlabel(ax2,'x(mm)')
ylabel(ax2,'Concentration')
end

function [aa,aa_upper,aa_lower]=increase(m_err,aa,aa_upper,aa_lower)
   if m_err>0
      aa_upper=aa;
   else
      aa_lower=aa;
   end
   aa=(aa_lower+aa_upper)/2;
end
```

References

[1] G. Freddi, A. Anghileri, S. Sampaio, J. Buchert, P. Monti, P. Taddei, *J. Biotechnol.* **2006**, 125, 281.
[2] M. McGill, G. P. Holland, D. L. Kaplan, *Macromol. Rapid Commun.* **2019**, 40, 1800390; X. Hu, D. Kaplan, P. Cebe, *Macromolecules* **2008**, 41, 3939.
[3] K. Tsioris, W. K. Raja, E. M. Pritchard, B. Panilaitis, D. L. Kaplan, F. G. Omenetto, *Adv. Func. Mater.* **2012**, 22, 330; C. E. Ghezzi, L. Wang, I. Behlau, J. Rnjak-Kovacina, S. Wang, M. H. Goldstein, J. Liu, J. K. Marchant, M. I. Rosenblatt, D. L. Kaplan, *J. Appl. Biomater. Func.* **2016**, 14, e266.
[4] Q. Lu, B. Zhang, M. Li, B. Zuo, D. L. Kaplan, Y. Huang, H. Zhu, *Biomacromolecules* **2011**, 12, 1080; D. J. Hines, D. L. Kaplan, *Biomacromolecules* **2011**, 12, 804.
[5] K. H. Jensen, K. Berg-Sørensen, H. Bruus, N. M. Holbrook, J. Liesche, A. Schulz, M. A. Zwieniecki, T. Bohr, *Rev. Mod. Phys.* **2016**, 88.
[6] E. W. Washburn, *Phys. Rev.* **1921**, 17, 273.
[7] S. Porfírio, M. D. G. da Silva, A. Peixe, M. J. Cabrita, P. Azadi, *Anal. Chim. Acta* **2016**, 902, 8.
[8] R. R. Jensen, S. S. Brake, S. F. Wolf, M. F. Bekker, P. J. Hardin, M. W. Jackson, *Environ. Earth Sci.* **2010**, 60, 1391.
[9] D. N. Rockwood, R. C. Preda, T. Yucel, X. Wang, M. L. Lovett, D. L. Kaplan, *Nat. Protoc.* **2011**, 6, 1612.
[10] B. Marelli, C. E. Ghezzi, A. Alessandrino, J. E. Barralet, G. Freddi, S. N. Nazhat, *Biomaterials* **2012**, 33, 102.
[11] X. Hu, D. Kaplan, P. Cebe, *Macromolecules* **2006**, 39, 6161.
[12] K. Nanjareddy, L. Blanco, M. K. Arthikala, X. A. Affantrange, F. Sánchez, M. Lara, *J. Integr. Plant Biol.* **2014**, 56, 281.
[13] M. C. White, A. M. Decker, R. L. Chaney, Plant Physiology 1981, 67, 292; J. Bialczyk, Z. Lechowsk, *J. Plant Nutr.* **1995**, 18, 2005.
